# Supplementary material for: Large-scale online assessment uncovers a distinct Multiple Sclerosis subtype with selective cognitive impairment
Source: Nat Commun. 2025 Sep 3;16:6938. doi: 10.1038/s41467-025-62156-4 (PMC12408825; doi:10.1038/s41467-025-62156-4)
Supplement: Supplementary file 1 — Supplementary Information [file 41467_2025_62156_MOESM1_ESM.pdf]

Supplementary Information

This appendix has been provided by the authors to give the reader additional information about their work.

Supplementary Figures

Figure S1. Sensitivity of accuracy deficits to outlier removal methods. Median (for the original and winsorisation methods) and mean (for the rank inverse transformation method) deviation from expected (mDfE) accuracy scores across tasks (N by task in Table S1) under different outlier removal methods, applied prior to regression-based normalisation. Error bars indicate 95% confidence intervals. Orange lines represent the control median. Pearson correlation coefficients and corresponding two-tailed p-values comparing mDfE accuracy scores for each alternative method with those from the original are reported in the subplot titles. Statistical significance of the DfE accuracy scores across tasks was assessed using two-tailed Wilcoxon signed-rank tests against zero for the original and winsorisation methods, and two-tailed t-tests against zero for the rank inverse transformation method. Resulting p-values are reported in the figure as: p>0.05=p, p≤0.05=*, p≤0.001=**, p≤0.0001=***. Source data are provided as a Source Data file.

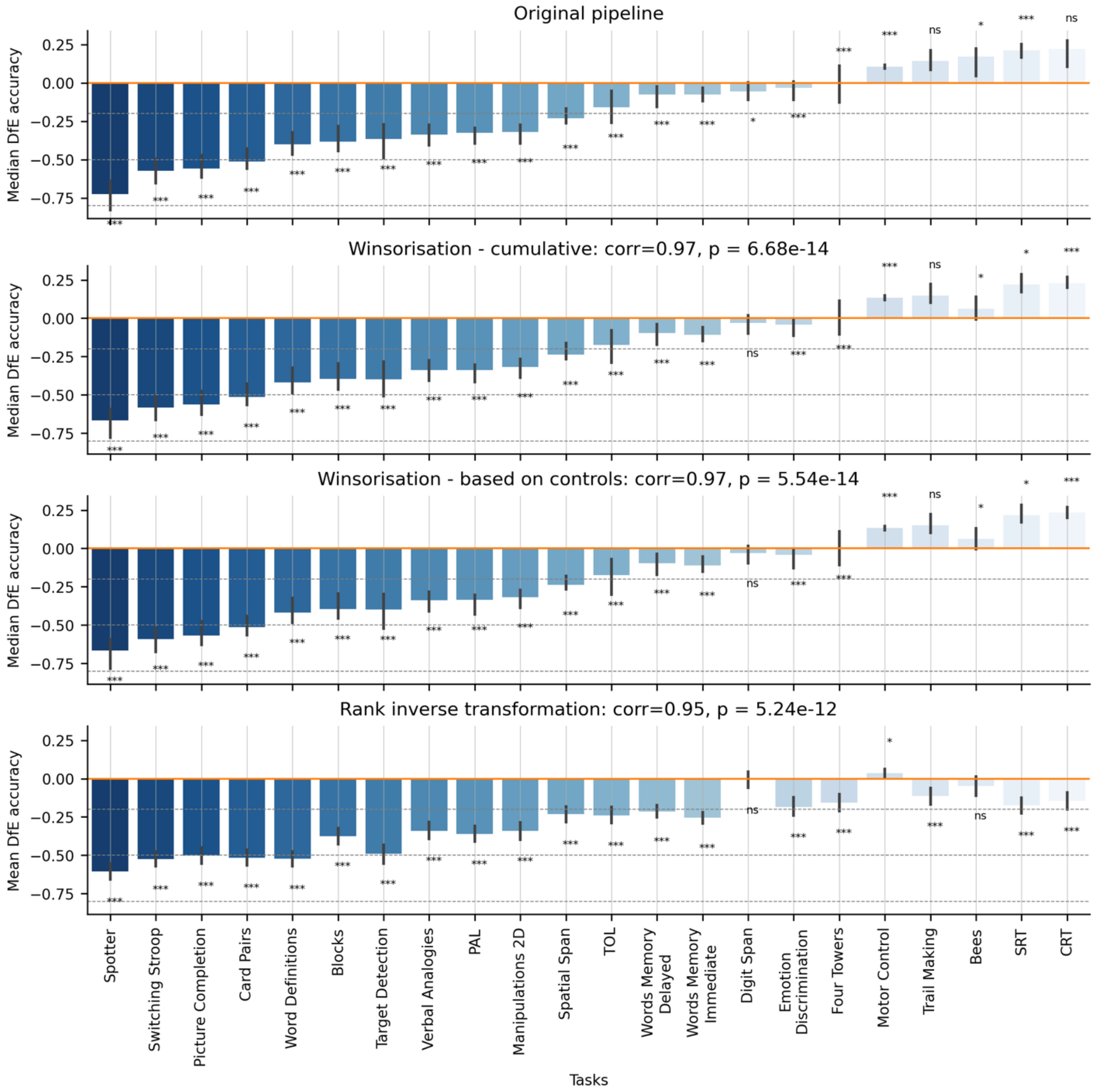

Figure S2. Sensitivity of response time (RT) deficits to outlier removal methods. Median (for the original and winsorisation methods) and mean (for the rank inverse transformation method) deviation from expected (mDfE) RTs across tasks (N by task in Table S1) under different outlier removal methods, applied prior to regression-based normalisation. Error bars indicate 95% confidence intervals. Orange lines represent the control median. Pearson correlation coefficients and corresponding two-tailed p-values comparing mDfE RTs for each alternative method with those from the original are reported in the subplot titles. Statistical significance of the DfE RTs across tasks was assessed using two-tailed Wilcoxon signed-rank tests against zero for the original and winsorisation methods, and two-tailed t-tests against zero for the rank inverse transformation method. Resulting p-values are reported in the figure as: p>0.05=p, p≤0.05=*, p≤0.001=**, p≤0.0001=***. Source data are provided as a Source Data file.

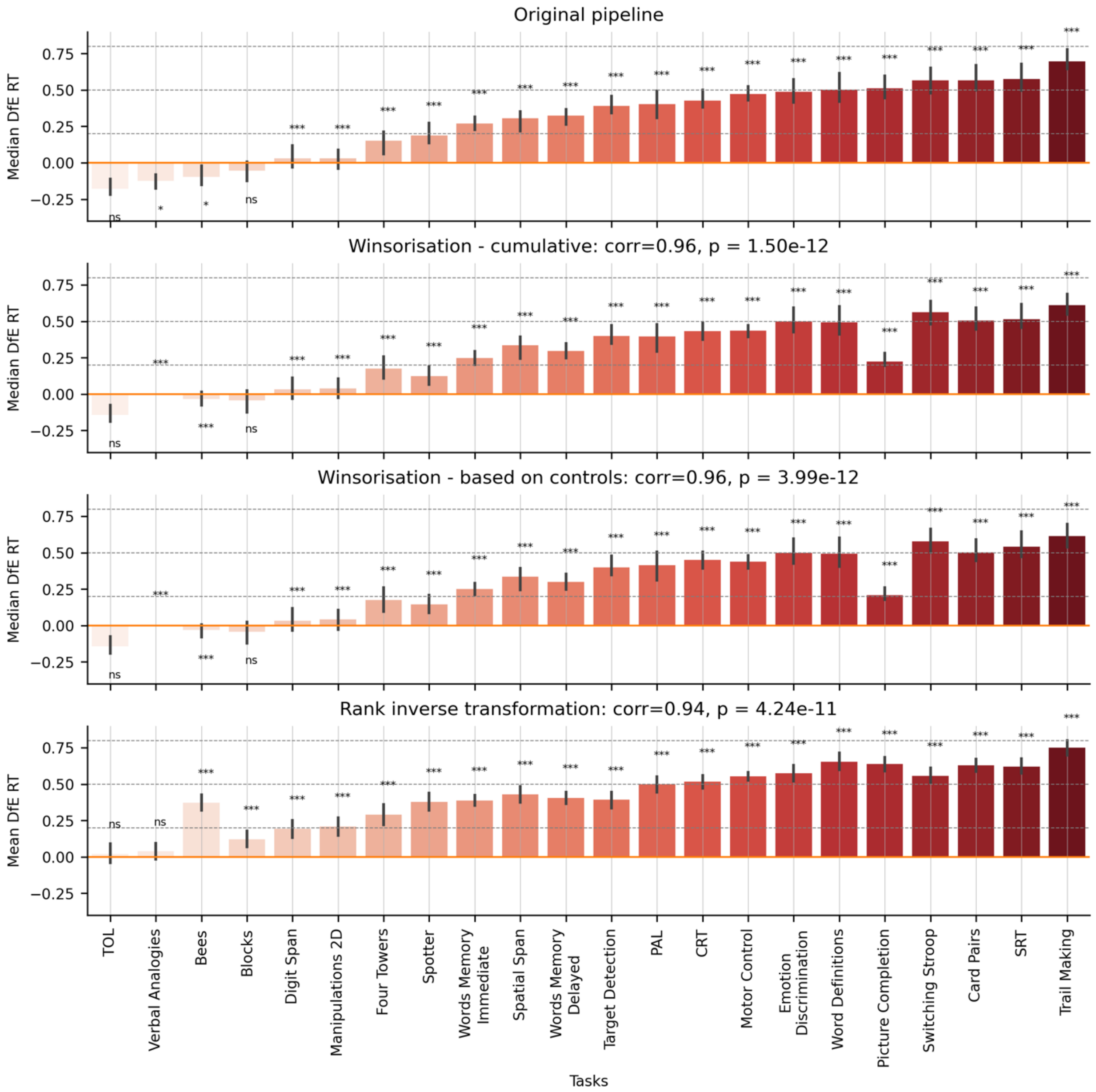

Figure S3. Sensitivity of accuracy and response time deficits to regression-based normalisation. Group differences between people with Multiple Sclerosis (pwMS; N by task in Table S1) and controls (N by task in Table S14) in **(A)** accuracy and **(B)** response time across tasks, evaluated either as Deviation from Expected (DfE) scores following regression-based normalisation or as standardised mean differences following propensity score matching. Error bars indicate 95% confidence intervals. Orange lines represent the control median. Pearson correlation coefficients and corresponding two-tailed p-values comparing group differences obtained using the two methods across tasks are reported in the subplot titles. Statistical significance of group differences across tasks was assessed using two-tailed Wilcoxon signed-rank tests against zero for the regression-based normalisation method, and two-tailed two-sample t-tests for the propensity matching method. Resulting p-values are reported in the figure as: p>0.05=p, p≤0.05=*, p≤0.001=**, p≤0.0001=***. Source data are provided as a Source Data file.

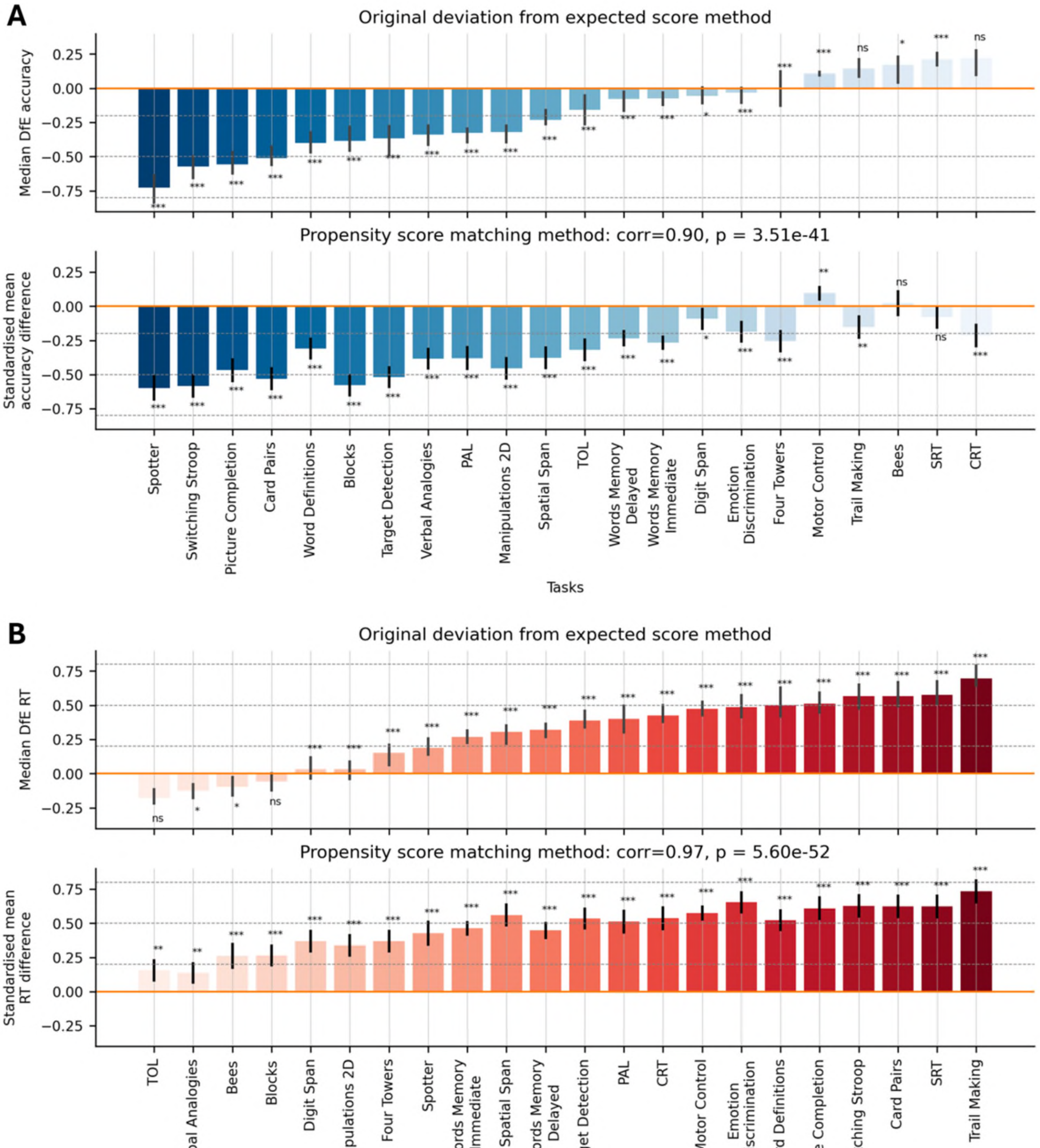
A
Original deviation from expected score method
Median DfE accuracy
Propensity score matching method: corr=0.90, p = 3.51e-41
Standardised mean accuracy difference
Spotter
Switching Stroop
Picture Completion
Card Pairs
Word Definitions
Blocks
Target Detection
Verbal Analogies
PAL
Manipulations 2D
Spatial Span
TOL
Words Memory Delayed
Words Memory Immediate
Digit Span
Emotion Discrimination
Four Towers
Motor Control
Trail Making
Bees
SRT
CRT
Tasks
B
Original deviation from expected score method
Median DfE RT
Propensity score matching method: corr=0.97, p = 5.60e-52
Standardised mean RT difference
TOL
Verbal Analogies
Bees
Blocks
Digit Span
Manipulations 2D
Four Towers
Spotter
Words Memory Immediate
Spatial Span
Words Memory Delayed
Target Detection
PAL
CRT
Motor Control
Emotion Discrimination
Word Definitions
Picture Completion
Switching Stroop
Card Pairs
SRT
Trail Making
Tasks

Figure S4. Scree plot of latent cognitive domains. Scree plot resulting from factor analysis of primary performance metrics across tasks from Stage 1 data (N by task in Table S1). 6 factors were selected as having eigenvalues > 1 (Kaiser's criterion).

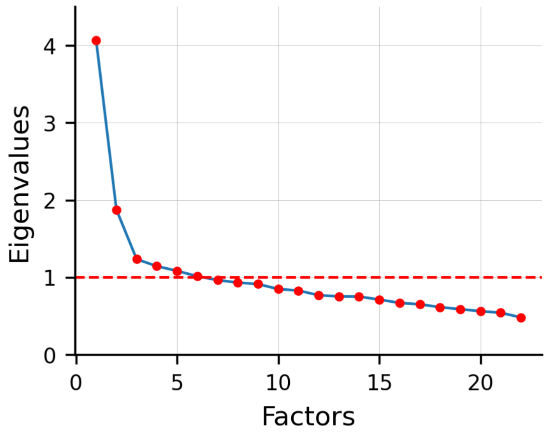

Figure S5. Split-half cross-validation of factor structure. Example comparison of factor loading matrices obtained by randomly splitting the dataset in two subgroups and performing factor analysis separately on each subgroup. Factor order was aligned using the Hungarian algorithm to maximise the average correlation between loadings.

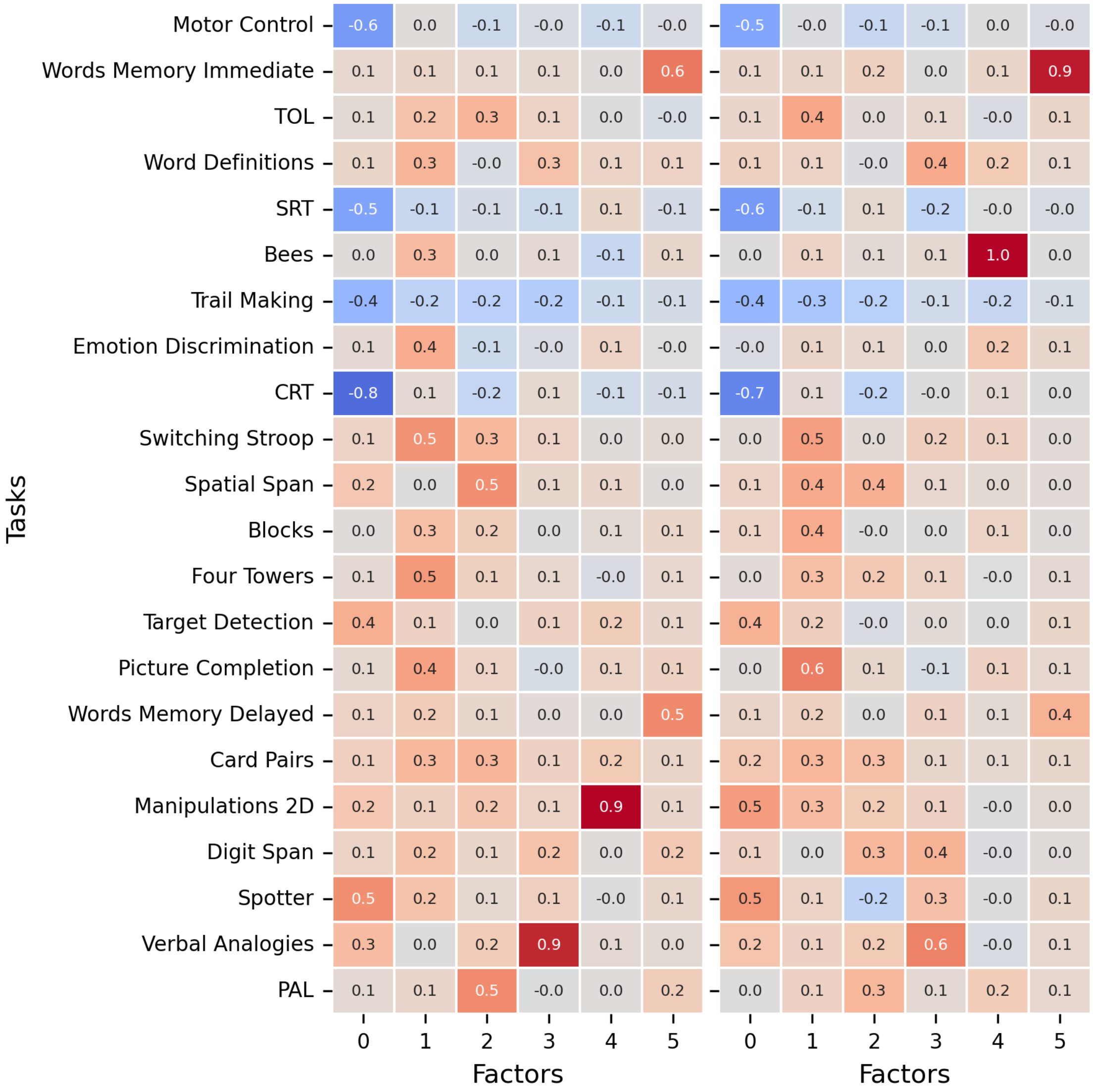

Figure S6. Stability of factor structure across sample sizes. Mean matched-factor correlations with 95% confidence intervals, estimated using a split-half validation procedure over 50 iterations, plotted as a function of sample size. Sample sizes tested ranged from 100 and 1,524 (i.e., up to half of the full dataset).

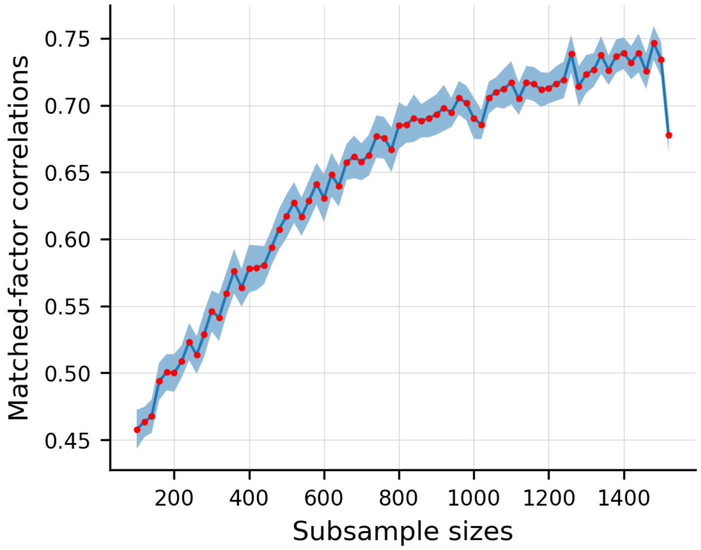

Figure S7. Device sensitivity for accuracy and response time metrics across tasks, evaluated on control data. Device sensitivity was defined as the eta squared resulting from a multiple linear regression model predicting each performance metric and including as predictors sociodemographic variables and device label. N by task is reported in Table S14. Values <0.06 are small and values <0.01 are considered negligible. Resulting p-values are reported in the figure as: p>0.05=p, p≤0.05=*, p≤0.001=**, p≤0.0001=***. Source data are provided as a Source Data file.

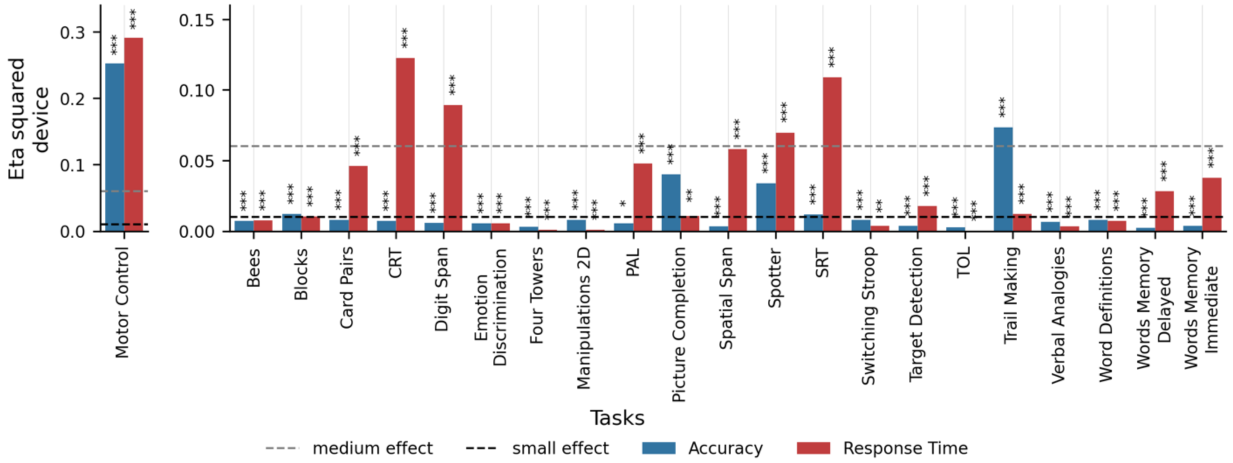

Figure S8. Pairwise comparisons of deviation from expected (DfE) scores across disease durations. P-values resulting from post-hoc pairwise comparisons carried out using the Mann-Whitney U test with Bonferroni correction for multiple comparisons of DfE scores across disease durations for performance metrics that showed a significant association with disease duration.

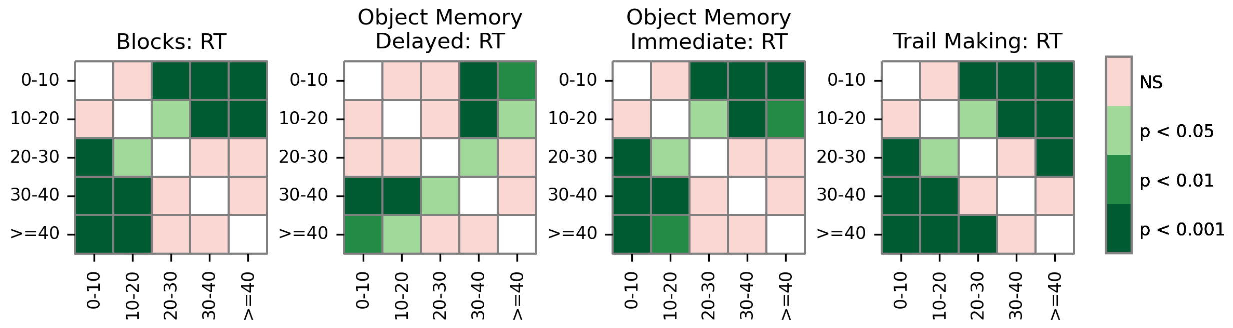

Figure S9. Pairwise comparisons of deviation from expected (DfE) scores across disease subtypes. P-values resulting from post-hoc pairwise comparisons carried out using the Mann-Whitney U test with Bonferroni correction for multiple comparisons of DfE scores across subtypes for performance metrics that showed a significant association with subtype.

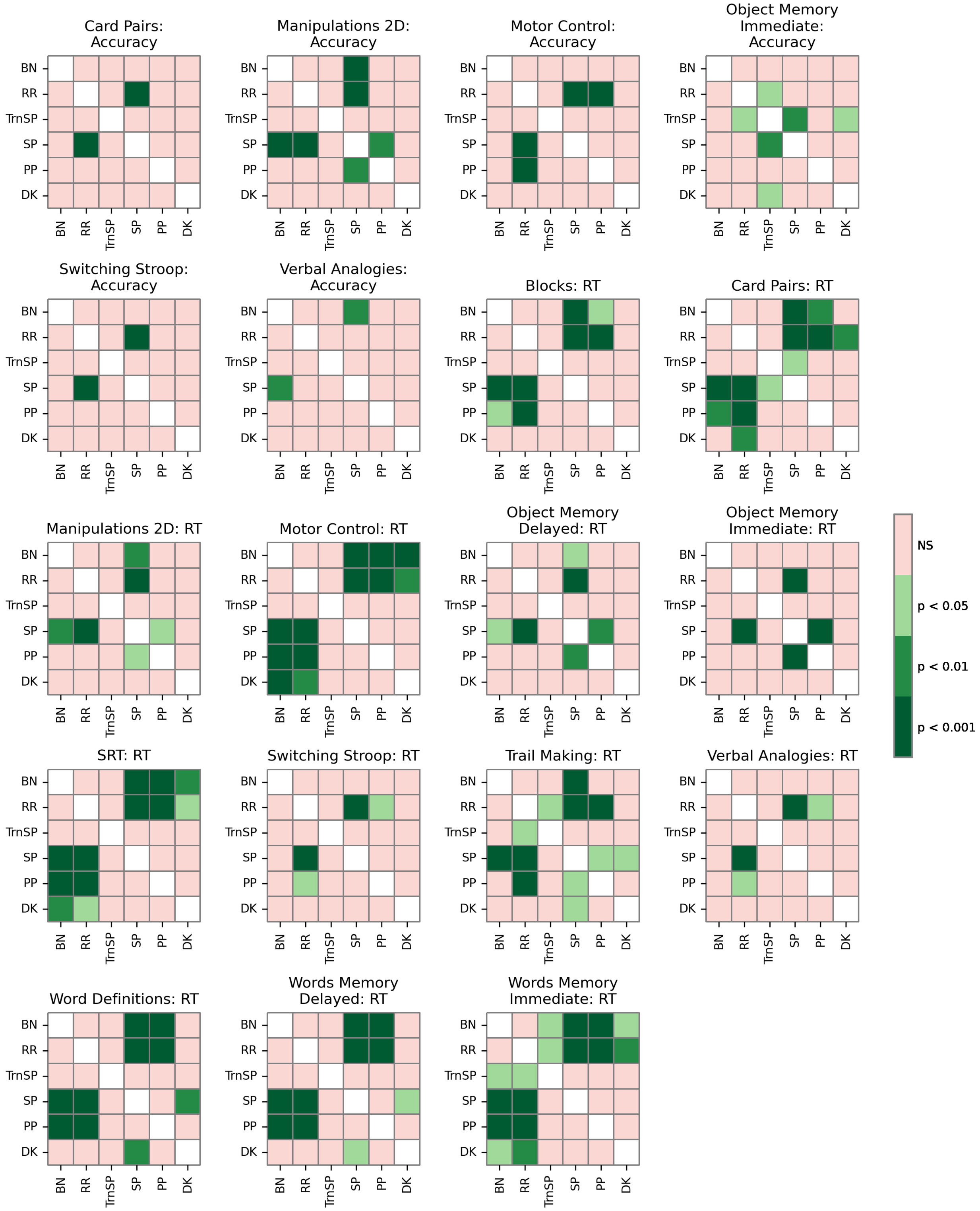

Figure S10. Clustering stability across methods, cluster numbers and similarity metrics. Stability curves for different similarity metrics evaluated across various clustering algorithms and cluster numbers. The cumulative score represents the primary stability metric used to evaluate and compare clustering solutions.

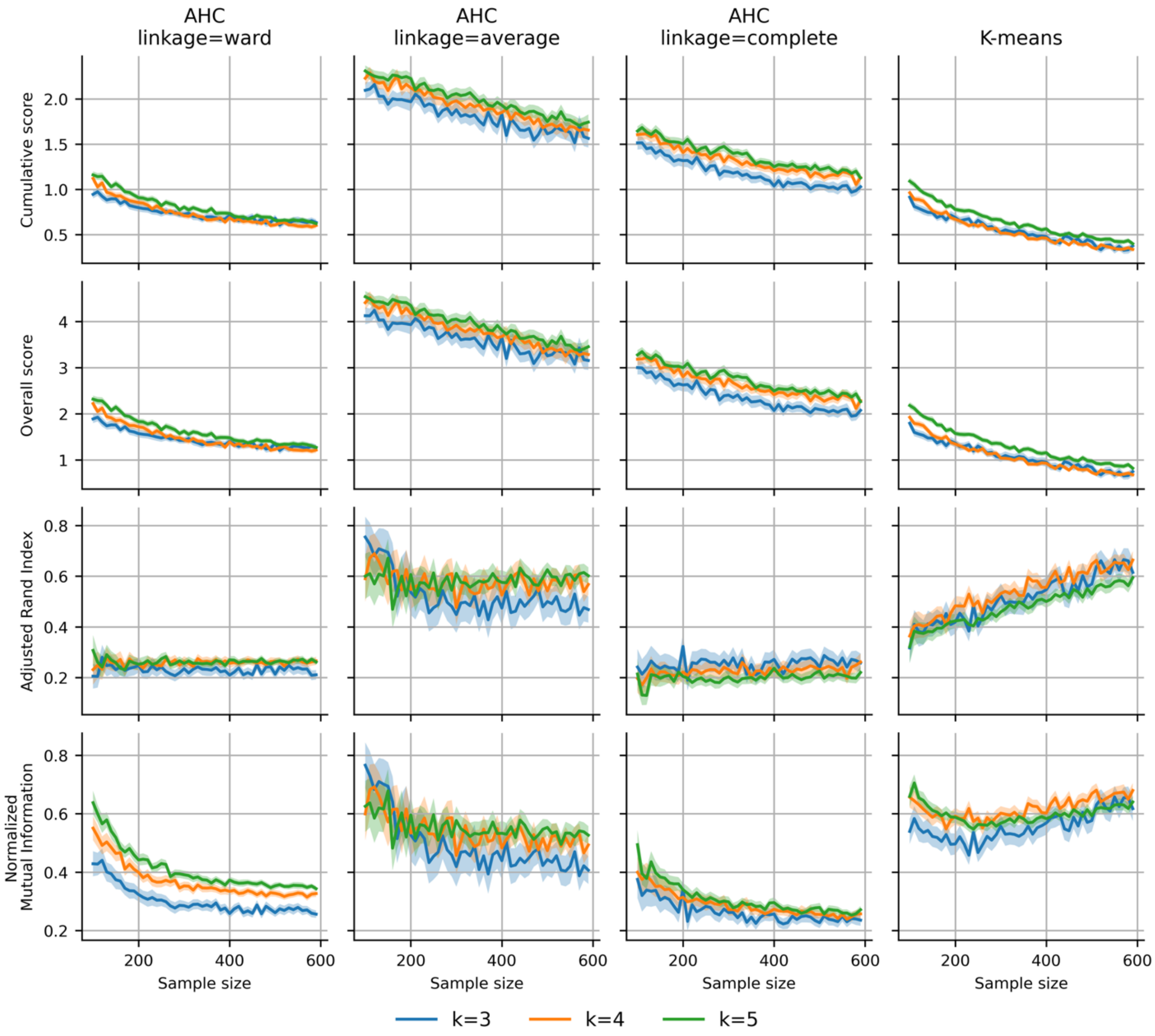

Figure S11. Clustering stability across methods, number of principal components selected and similarity metrics. Stability curves for different similarity metrics evaluated across a range of clustering algorithms and number of components selected as input features following dimensionality reduction via principal component analysis (PCA). The number of clusters was fixed at 4.

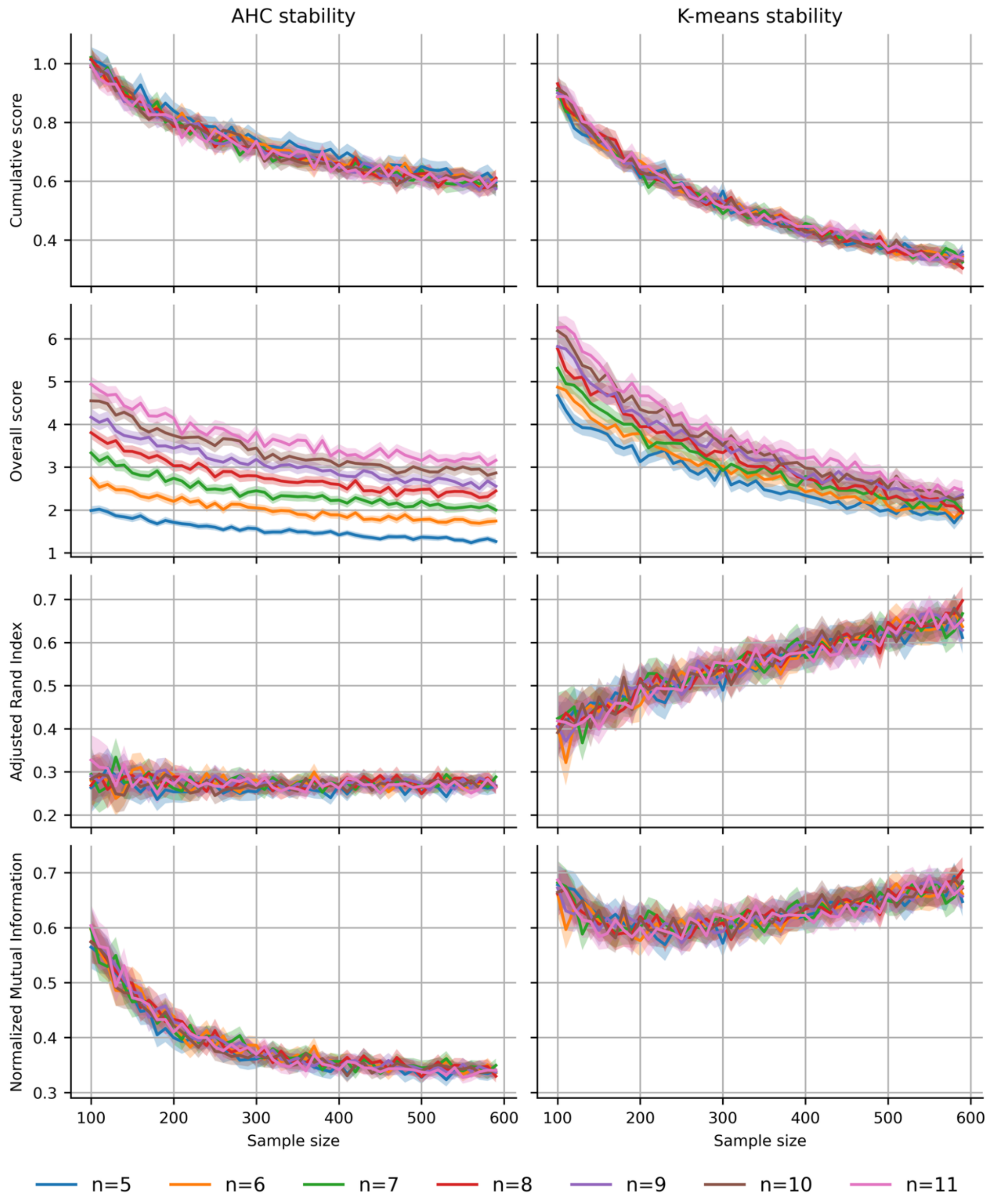

Figure S12. Clustering stability across methods with and without dimensionality reduction. Stability curves for different similarity metrics evaluated using two clustering algorithms, with or without applying principal component analysis (PCA) for input dimensionality reduction. The number of clusters was fixed at 4 and 5 principal components were selected when PCA was applied.

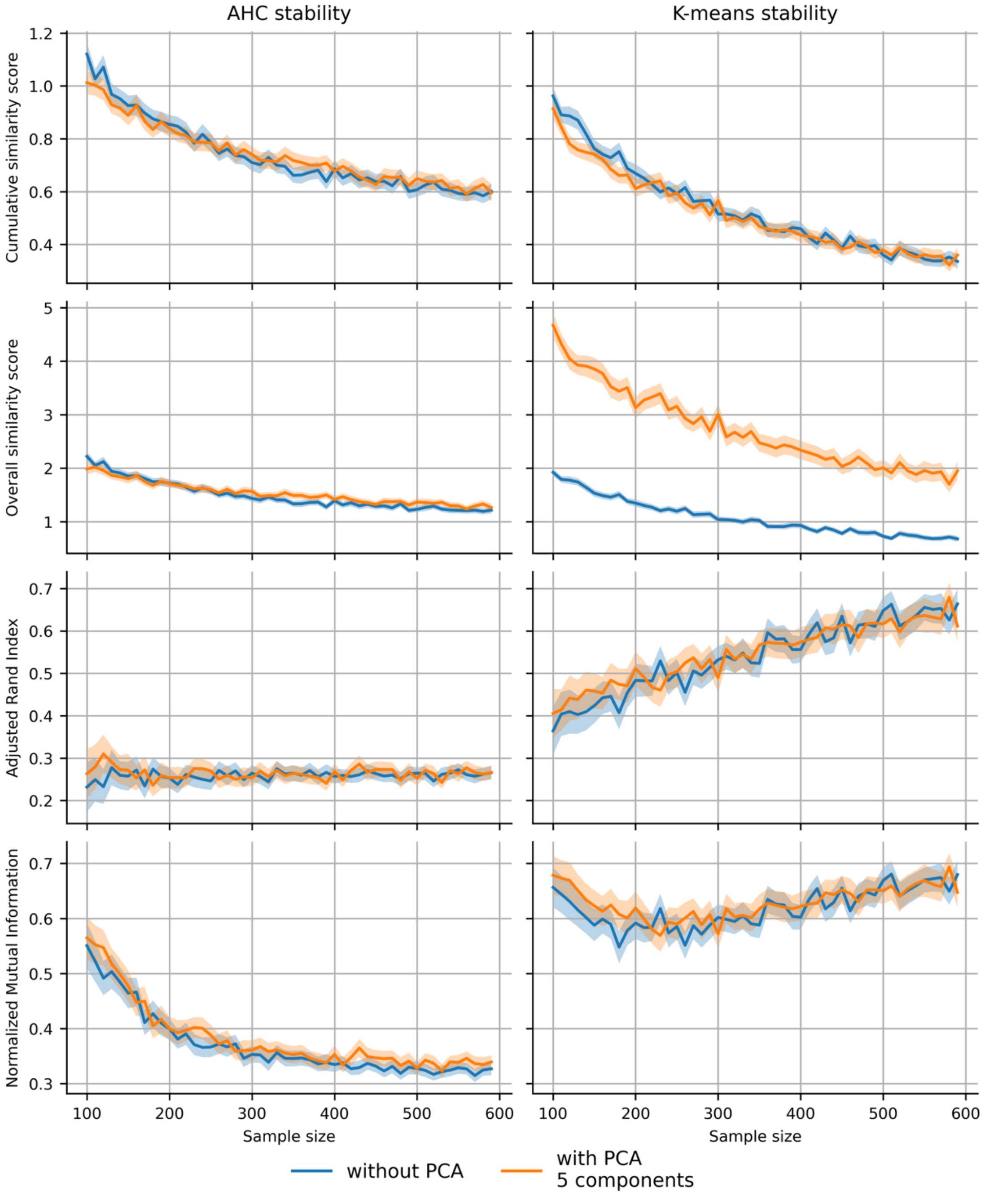

Figure S13. Pairwise comparisons of deviation from expected (DfE) scores and MSIS-29 motor and MSWS-12 scores across clusters. P-values resulting from post-hoc pairwise comparisons carried out using Dunn's tests with Bonferroni correction for multiple comparisons of DfE scores and MSIS-29 motor and MSWS-12 scores across clusters.

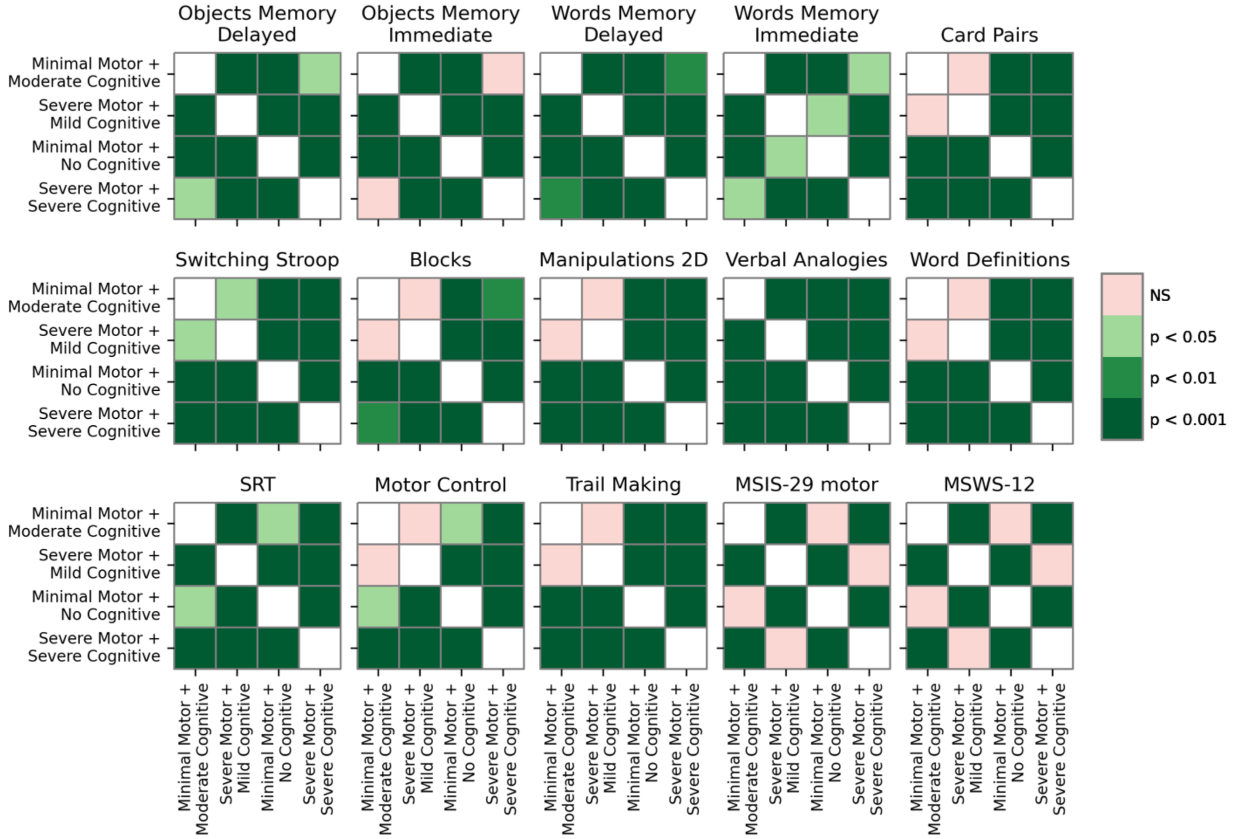

Figure S14. Pairwise comparisons of non-clustering patient-reported outcome (PRO) scores across clusters. P-values resulting from post-hoc pairwise comparisons carried out using Dunn's tests with Bonferroni correction for multiple comparisons of HADS-A, HADS-D, FSS, EQ5D, and webEDSS scores across clusters.

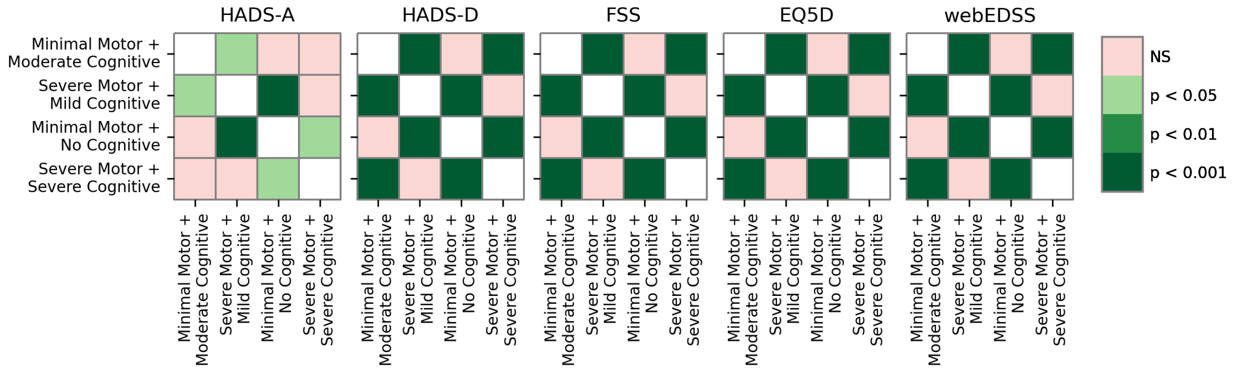

Figure S15. Impact of task modifications on performance. Standardised regression coefficients for task modifications, resulting from multiple linear regression models predicting performance scores across tasks from sociodemographic variables, device type, and task modifications. Red dotter lines indicate the threshold for small, non-negligible effect sizes. Significant standardised regression coefficients (p value ≤ 0.05) are reported in yellow. Source data are provided as a Source Data file.

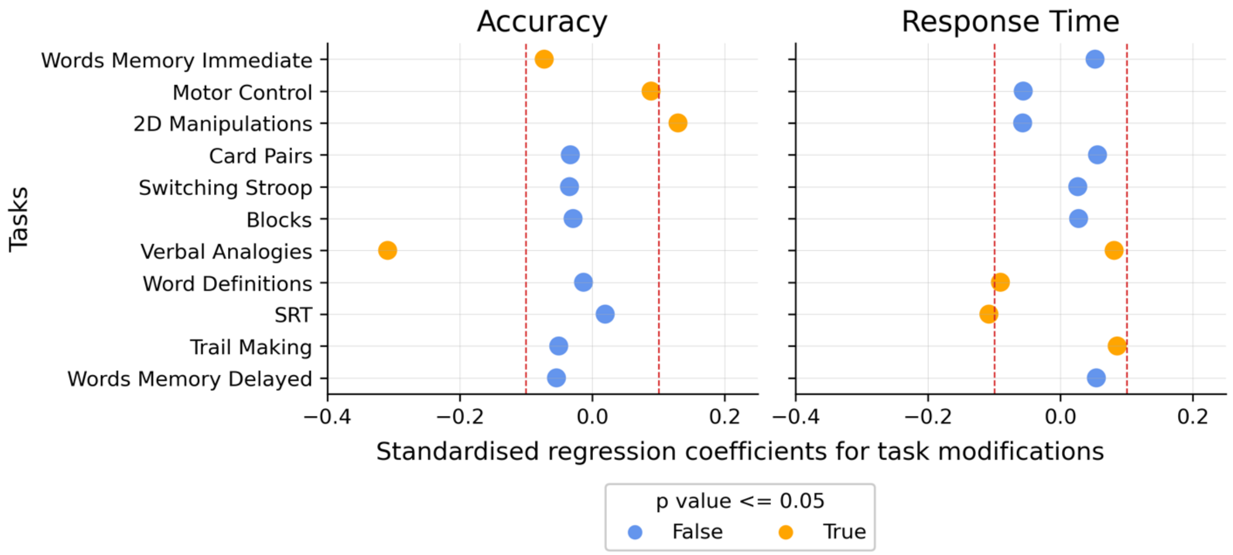

Figure S16. Uncorrected pairwise comparisons of deviation from expected (DfE) scores across disease durations. P-values resulting from post-hoc pairwise comparisons carried out using the Mann-Whitney U test without correcting for multiple comparisons of DfE scores across disease durations for performance metrics that showed a significant association with disease duration.

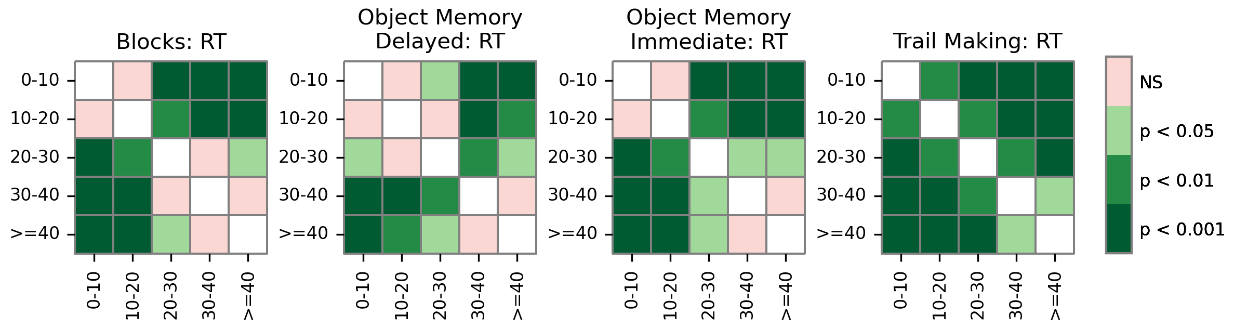

Figure S17. Uncorrected pairwise comparisons of deviation from expected (DfE) score across disease subtypes. P-values resulting from post-hoc pairwise comparisons carried out using the Mann-Whitney U test without correcting for multiple comparisons of DfE scores across subtypes for performance metrics that showed a significant association with subtype.

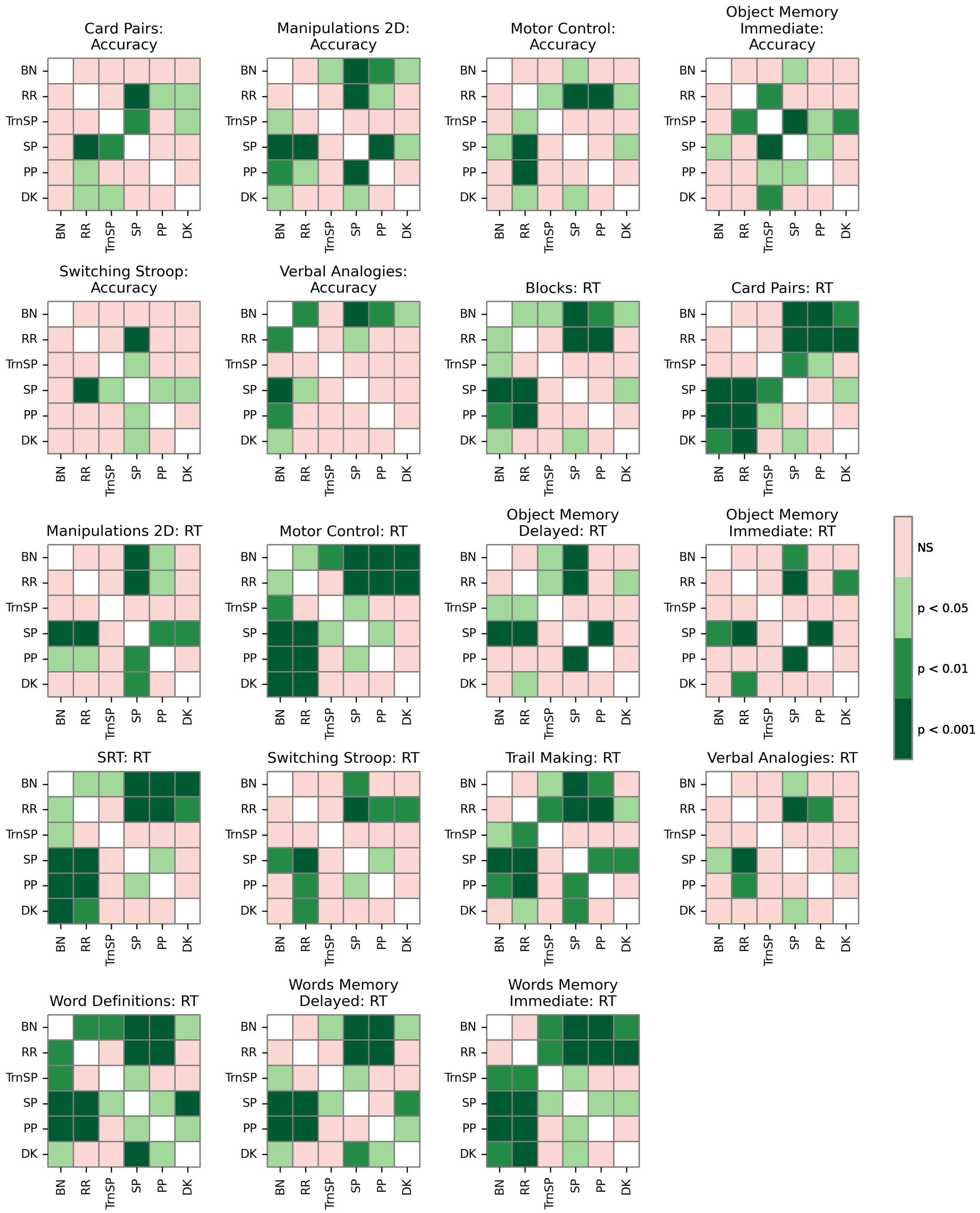

Figure S18. Uncorrected pairwise comparisons of deviation from expected (DfE) scores and MSIS-29 motor and MSWS-12 scores across clusters. P-values resulting from post-hoc pairwise comparisons carried out using Dunn's tests without correcting for multiple comparisons of DfE scores and MSIS-29 motor and MSWS-12 scores across clusters.

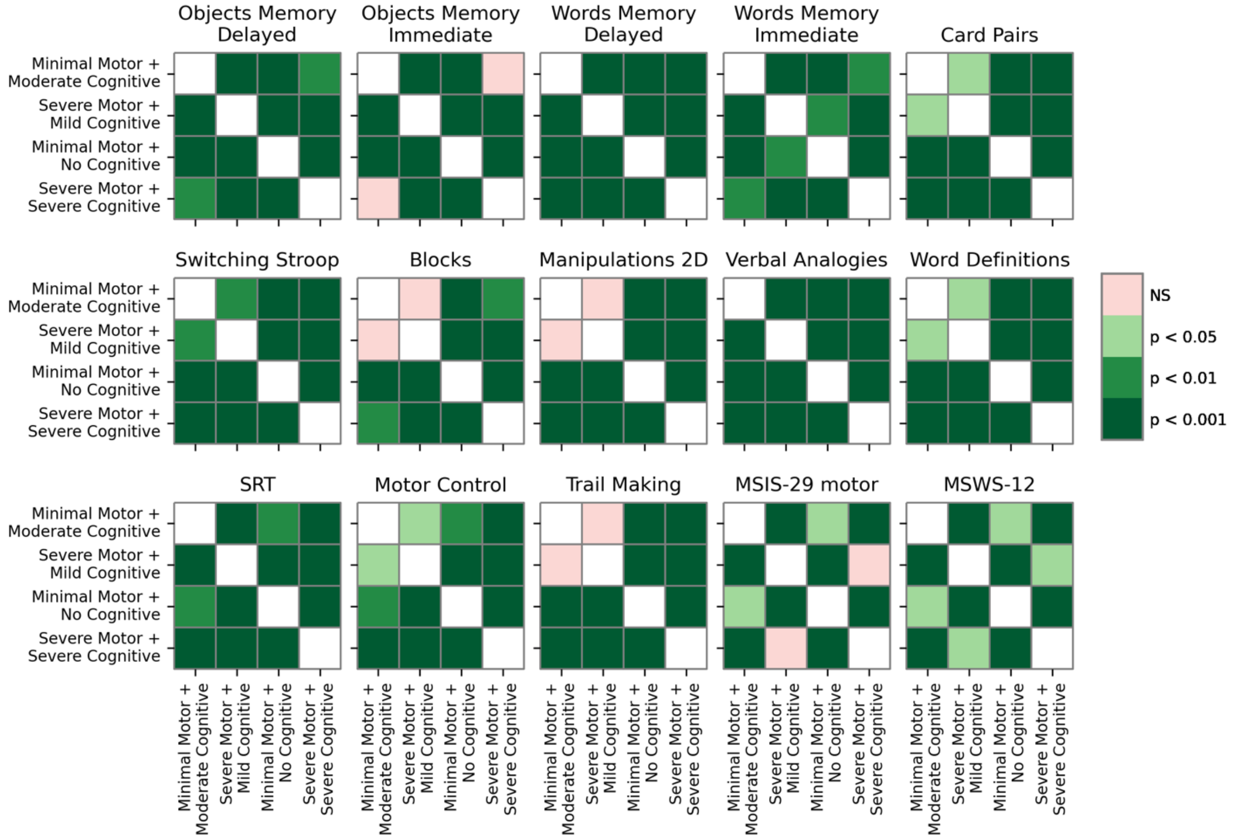

Figure S19. Uncorrected pairwise comparisons of non-clustering patient-reported outcome (PRO) scores across clusters. P-values resulting from post-hoc pairwise comparisons carried out using Dunn's tests without correcting for multiple comparisons of HADS-A, HADS-D, FSS, EQ5D and webEDSS scores across clusters.

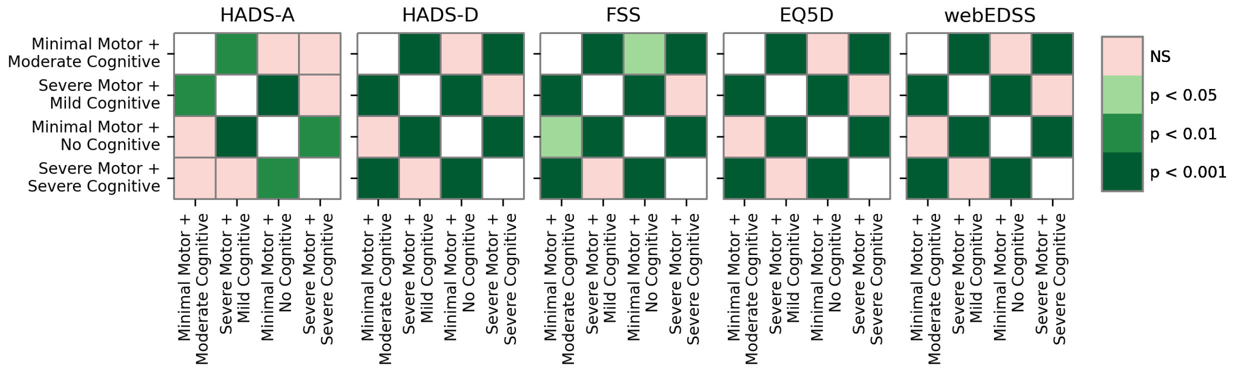

Figure S20. Distribution of task performance metrics across Stage 1 participants. Violin plots showing the distribution of task performance metrics across Stage 1 participants (N by task in Table S1). The width of each violin represents the kernel density estimate of the data distribution. The red centre line indicates the median; the two blue lines denote the first and third quartiles. Each violin represents one task metric.

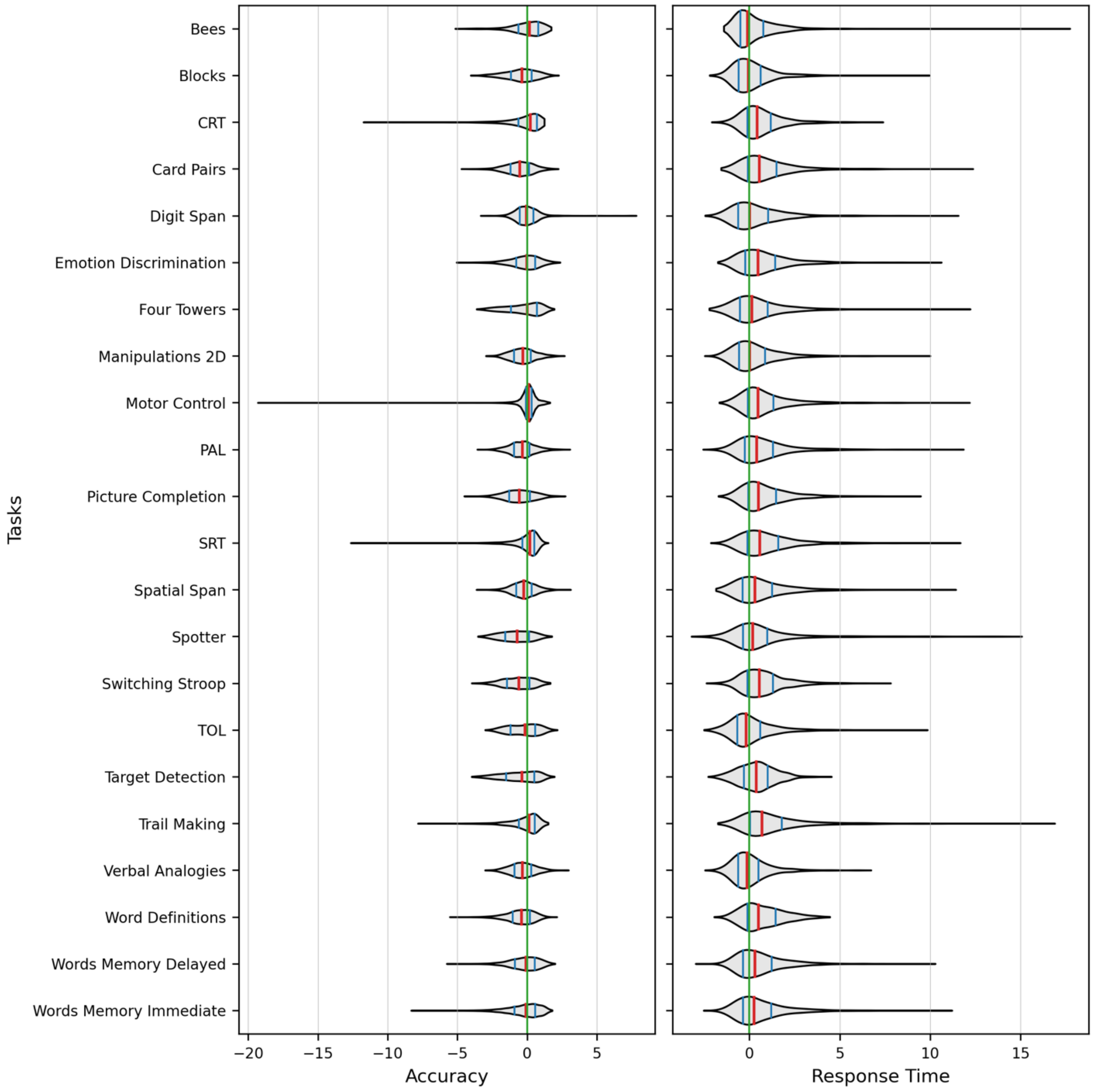

Figure S21. Distribution of task performance metrics across Stage 2 participants. Violin plots showing the distribution of task performance metrics across Stage 2 participants (N by task in Table S2). The width of each violin represents the kernel density estimate of the data distribution. The red centre line indicates the median; the two blue lines denote the first and third quartiles. Each violin represents one task metric.

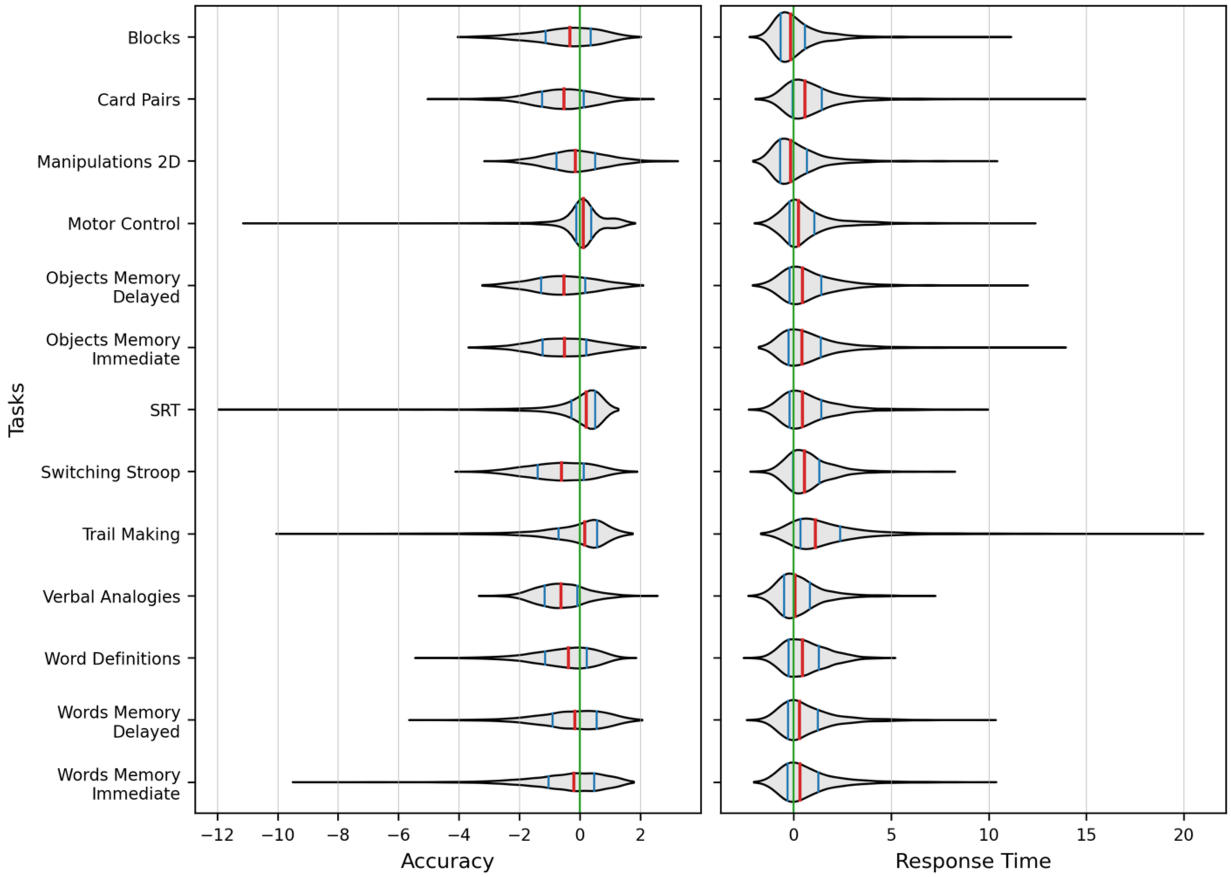

Figure S22. Distribution of accuracy performance metrics across subtypes in Stage 2 participants. Violin plots showing the distribution of accuracy metrics across subtypes in Stage 2 participants (N by task in Table S2). The width of each violin represents the kernel density estimate of the data distribution. The red centre line indicates the median; the two dotted black lines denote the first and third quartiles.

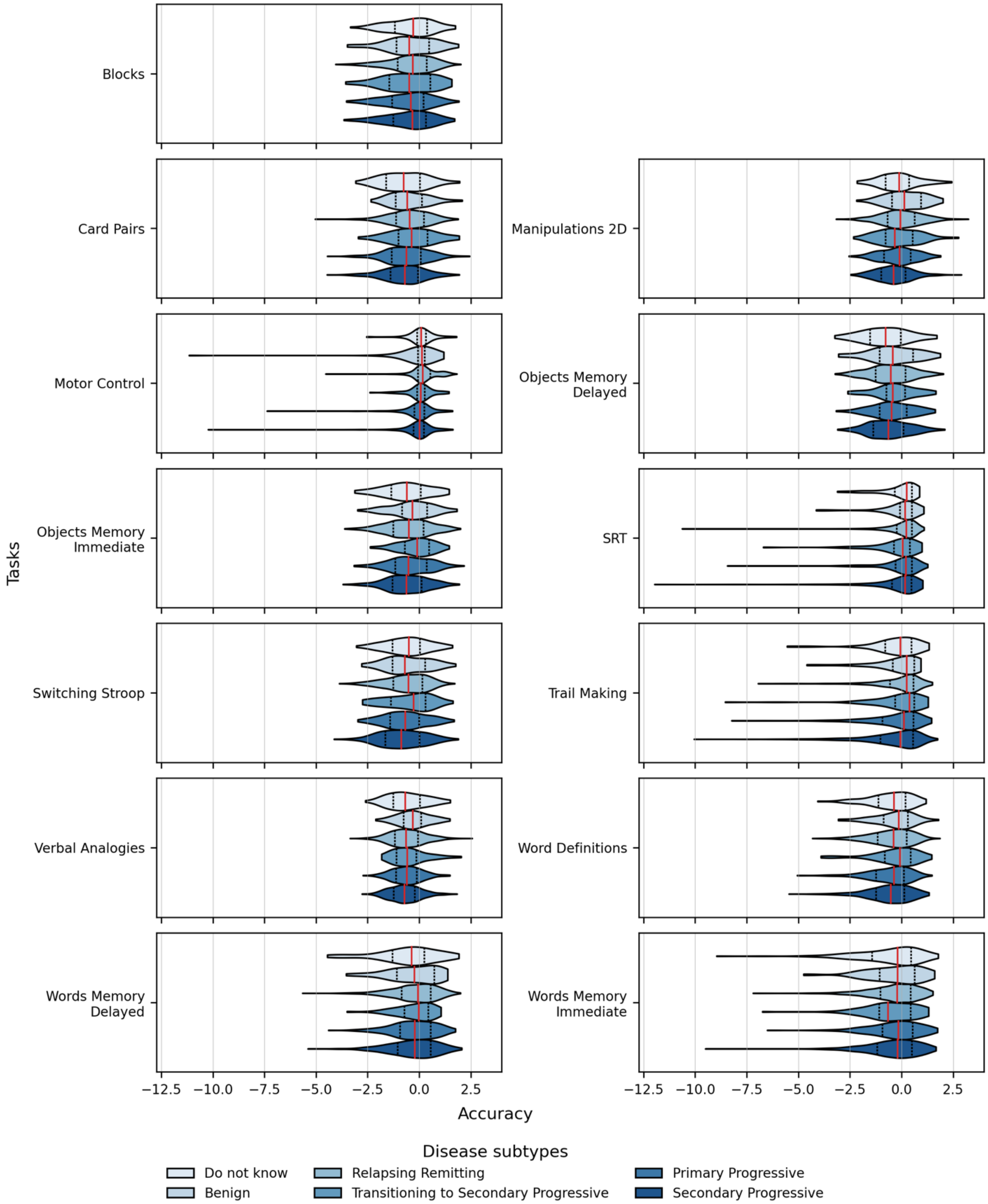

Figure S23. Distribution of response time metrics across subtypes in Stage 2 participants. Violin plots showing the distribution of response times across subtypes in Stage 2 participants (N by task in Table S2). The width of each violin represents the kernel density estimate of the data distribution. The red centre line indicates the median; the two dotted black lines denote the first and third quartiles.

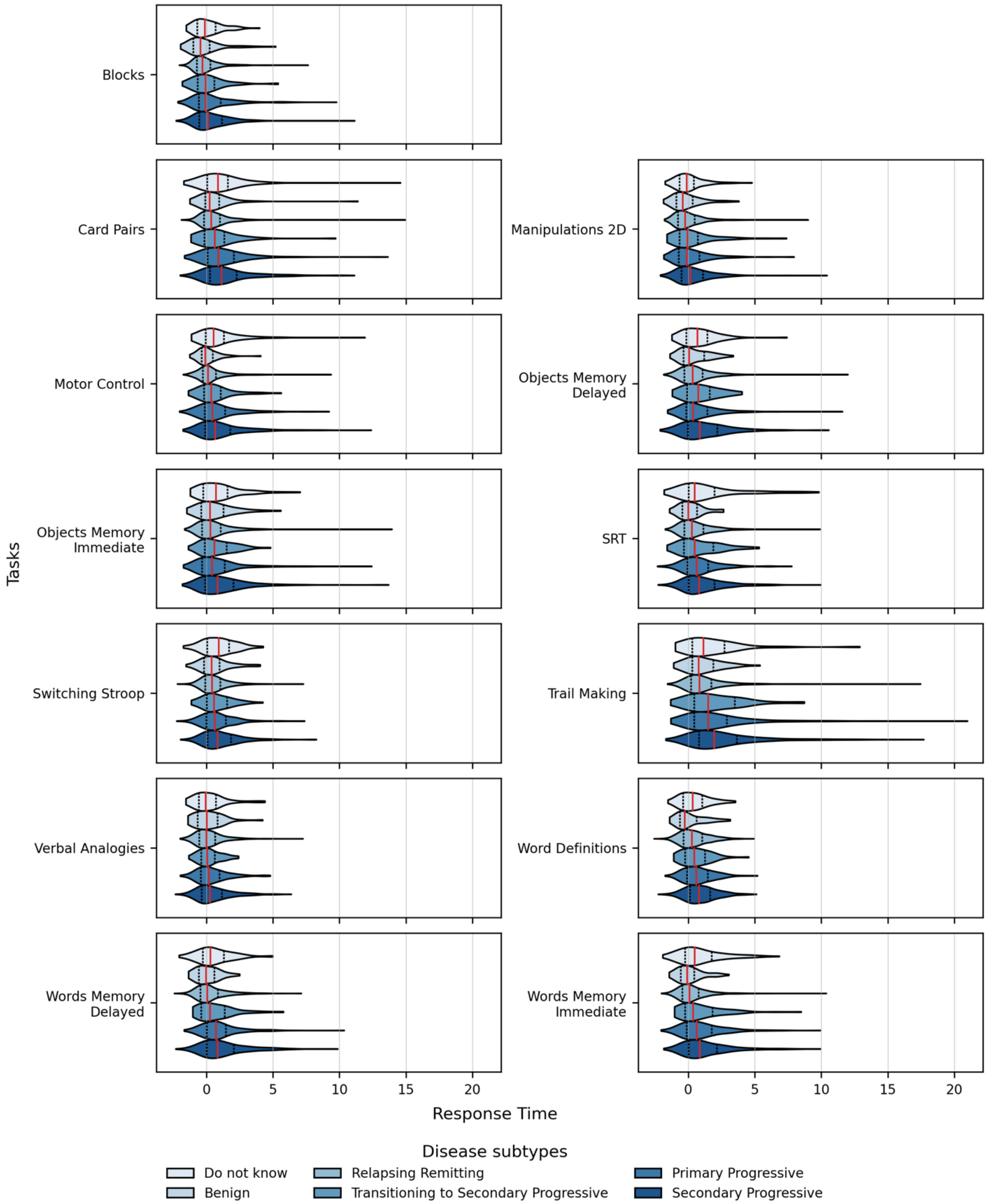

Figure S24. Distribution of accuracy performance metrics across disease durations in Stage 2 participants. Violin plots showing the distribution of accuracy metrics across disease durations in Stage 2 participants (N by task in Table S1). The width of each violin represents the kernel density estimate of the data distribution. The black centre line indicates the median; the two dotted black lines denote the first and third quartiles.

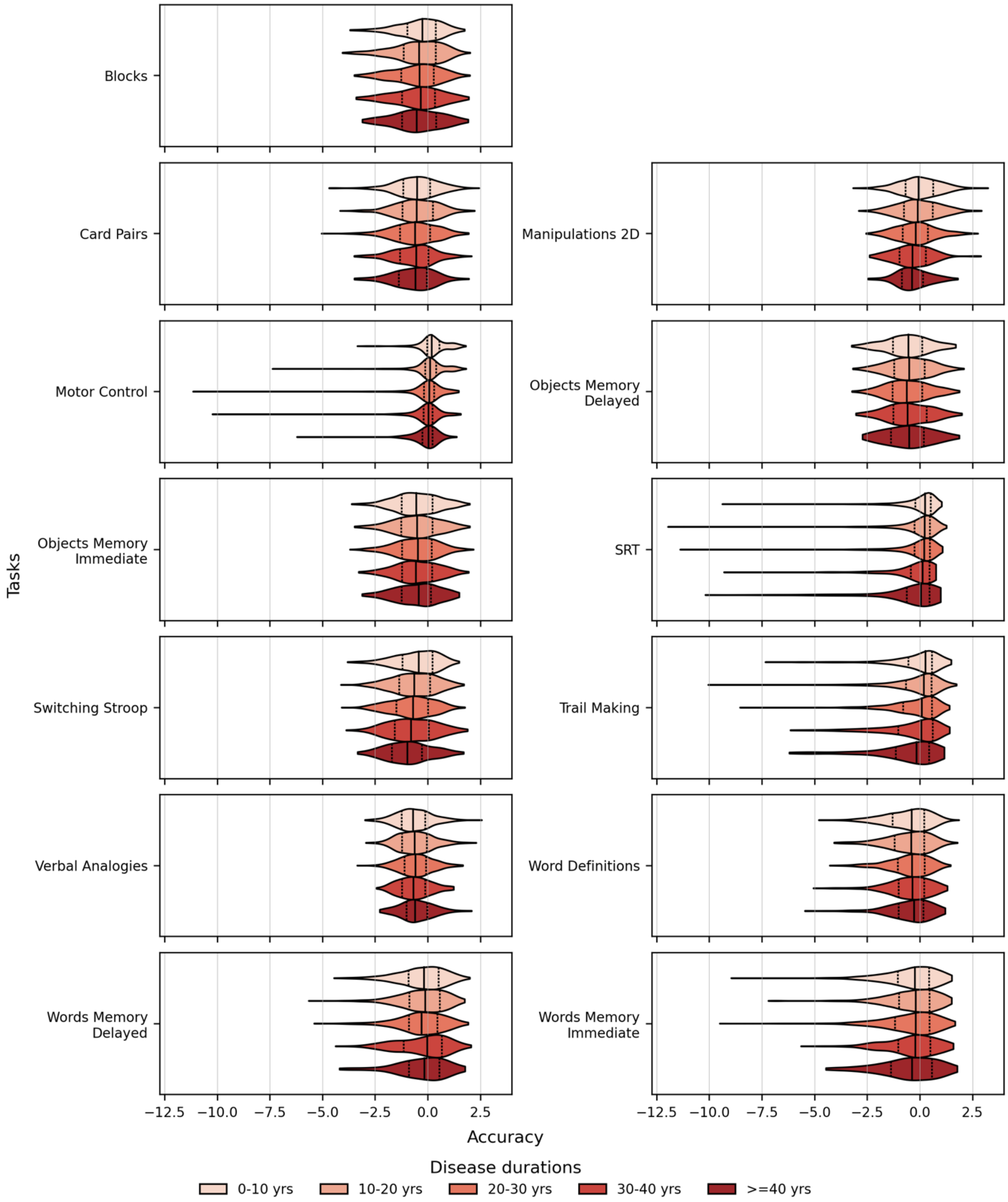

Figure S25. Distribution of response time metrics across disease durations in Stage 2 participants. Violin plots showing the distribution of response times across disease durations in Stage 2 participants (N by task in Table S1). The width of each violin represents the kernel density estimate of the data distribution. The black centre line indicates the median; the two dotted black lines denote the first and third quartiles.

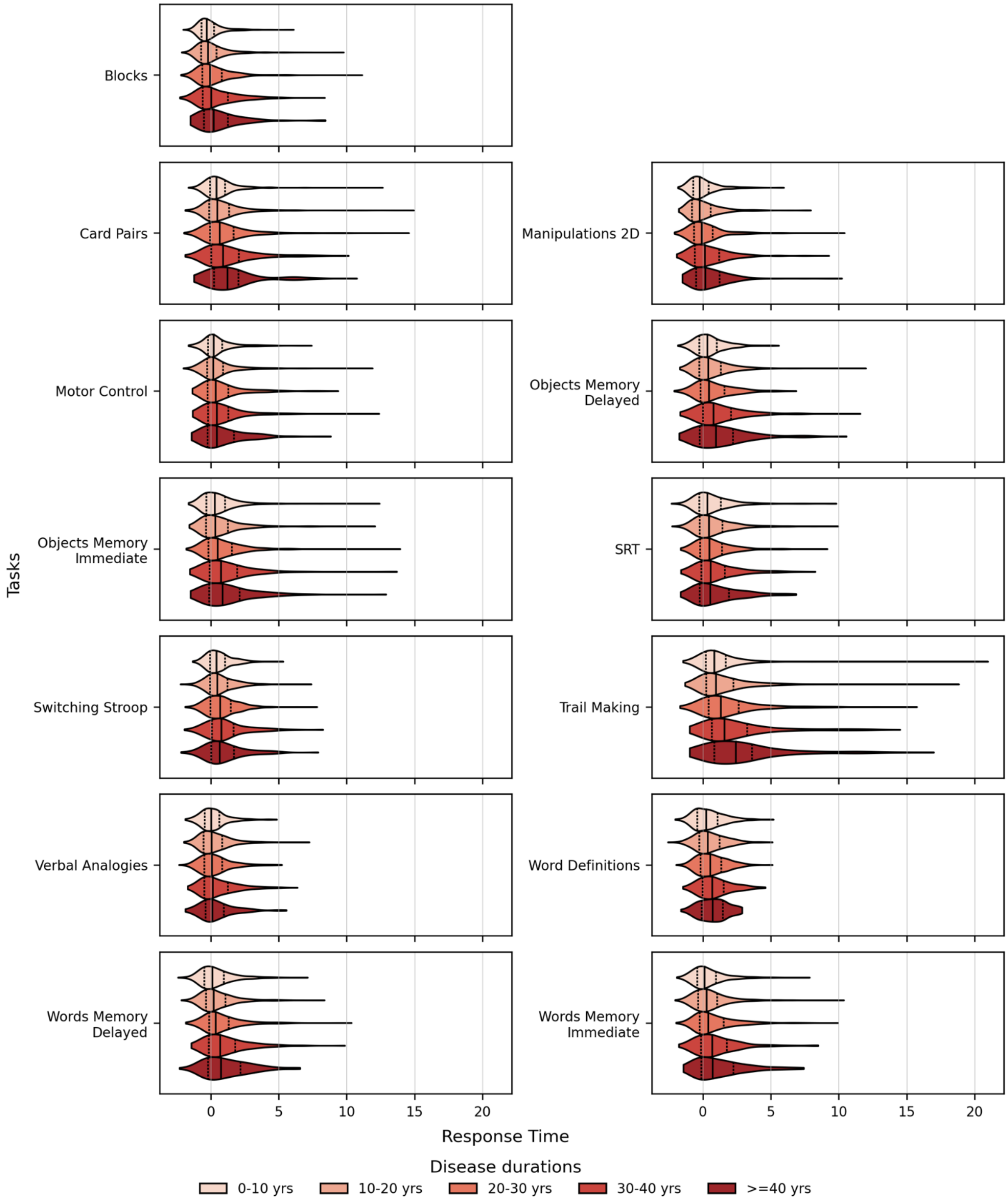

Figure S26. Distribution of task performance metrics across clusters in Stage 2 participants. Violin plots showing the distribution of task performance metrics across clusters in Stage 2 participants who completed the cognitive assessment as well as the MSIS-29 and MSWS-12 (N=1,180). The width of each violin represents the kernel density estimate of the data distribution. The black lines indicate the median and the first and third quartiles.

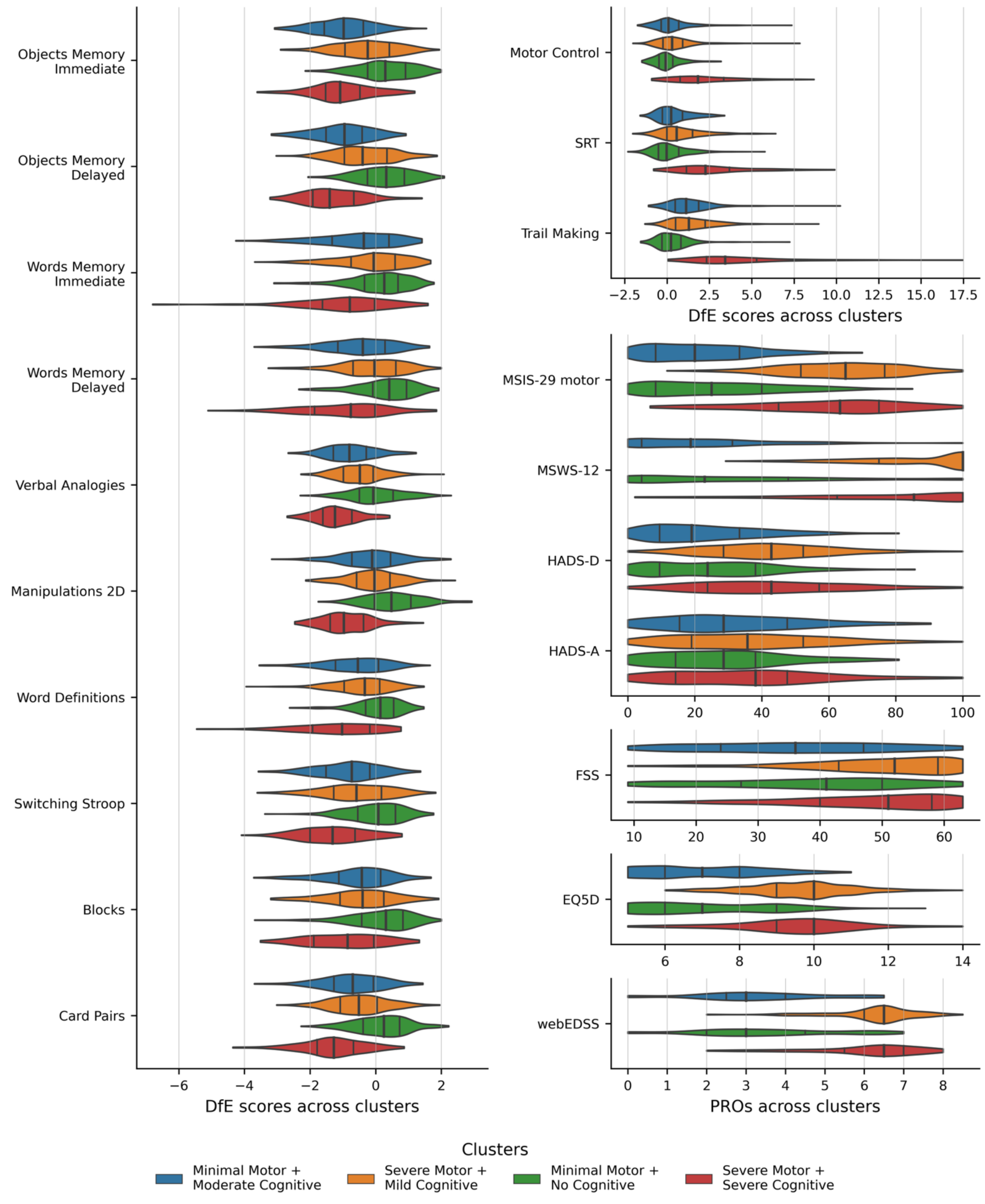
Objects Memory Immediate
Objects Memory Delayed
Words Memory Immediate
Words Memory Delayed
Verbal Analogies
Manipulations 2D
Word Definitions
Switching Stroop
Blocks
Card Pairs
−6 −4 −2 0 2
DfE scores across clusters
Motor Control
SRT
Trail Making
−2.5 0.0 2.5 5.0 7.5 10.0 12.5 15.0 17.5
DfE scores across clusters
MSIS-29 motor
MSWS-12
HADS-D
HADS-A
0 20 40 60 80 100
FSS
10 20 30 40 50 60
EQ5D
6 8 10 12 14
webEDSS
0 1 2 3 4 5 6 7 8
PROs across clusters
Clusters
Minimal Motor + Moderate Cognitive
Severe Motor + Mild Cognitive
Minimal Motor + No Cognitive
Severe Motor + Severe Cognitive

Supplementary Tables

Table S1. Stage 1 data availability. Number of participants who completed each task at Stage 1 before and after preprocessing procedures were applied.

Task	N participants (before preprocessing)	N participants (after preprocessing)
Sociodemographic questionnaire	3162	3155
Bees	1111	960
Blocks	1223	1213
Card Pairs	1235	1227
CRT	1264	1180
Digit Span	1173	1136
Emotion Discrimination	1210	1200
Four Towers	1198	1157
Manipulations 2D	1187	1169
Motor Control	3065	3029
PAL	1206	1196
Picture Completion	1242	1234
Spatial Span	1151	1130
Spotter	1110	1071
SRT	1233	1194
Switching Stroop	1252	1229
Target Detection	1268	1246
TOL	1212	1152
Trail Making	1207	1197
Verbal Analogies	1242	1225
Word Definitions	1241	1223
Words Memory Delayed	2412	2222
Words Memory Immediate	3021	2980

Table S2. Stage 2 data availability. Number of participants who completed each task at Stage 2 before and after preprocessing procedures were applied and number of participants performing the assessment for the first time across each task (independent sample).

Task	N participants (before preprocessing)	N participants (after preprocessing)	N participants – independent sample (after preprocessing)
Sociodemographic questionnaire	2891	2885	1426
Blocks	2393	2381	1232
Card Pairs	2519	2499	1310
Manipulations 2D	2576	2557	1349
Motor Control	2611	2597	1370
Objects Memory Delayed	2265	1899	976
Objects Memory Immediate	2696	2681	1421
Spotter	2197	-	-
SRT	2327	2263	1169
Switching Stroop	2471	2439	1268
Trail Making	2288	2275	1164
Verbal Analogies	2372	2331	1200
Word Definitions	2339	2324	1195
Words Memory Delayed	2252	1927	991
Words Memory Immediate	2632	2621	1382

Table S3. People with Multiple Sclerosis (MS) sociodemographic characteristics Stage 1. Sociodemographic characteristics of people with MS from the UK MS Register who participated in Stage 1 and were retained for analysis after preprocessing procedures were applied.

Sociodemographic characteristics		Participants
Total N		3048
Age (min-max, mean)		16-87, 54.4
Sex	Female	2359 (77.4%)
	Male	682 (22.4%)
	Other	7 (0.2%)
Dominant hand	Right	2653 (87%)
	Left	320 (10.5%)
	Ambidextrous	75 (2.5%)
First language	English	2934 (96.3%)
	Other	114 (3.7%)
Ethnicity	White	2888 (94.8%)
	Unknown	59 (1.9%)
	Mixed or multiple ethnic groups	42 (1.4%)
	Asian or Asian British	35 (1.1%)
	Black, Black British, Caribbean or African	22 (0.7%)
	American Hispanic	2 (0.1%)
Residence	United Kingdom	3025 (99.2%)
	Abroad	23 (0.8%)
Education	preGCSE	44 (1.4%)
	High School	1394 (45.7%)
	University Degree	1515 (49.7%)
	PhD	95 (3.1%)
Occupation	Worker	1232 (40.4%)
	Retired	1117 (36.6%)
	Disabled / Not applicable / Sheltered employment	492 (16.1%)
	Homemaker	125 (4.1%)
	Unemployed / Looking for work	53 (1.7%)
	Student	21 (0.7%)
	Unknown	8 (0.3%)
Disease subtype	Benign	53 (2.2%)
	Relapsing-remitting	1268 (53.8%)
	Secondary progressive	704 (29.9%)
	Primary progressive	331 (14%)
	missing = 692	
Disease duration (min-max, mean)		0-67, 20.4 (missing = 692)

Table S4. People with Multiple Sclerosis (MS) sociodemographic characteristics Stage 2. Sociodemographic characteristics of people with MS from the UK MS Register who participated in Stage 2 and were retained for analysis after preprocessing procedures were applied.

Sociodemographic characteristics		Participants
Total N		2690
Age (min-max, mean)		18-88, 53.9
Sex	Female	2099 (78%)
	Male	589 (21.9%)
	Other	2 (0.1%)
Dominant hand	Right	2315 (86.1%)
	Left	306 (11.4%)
	Ambidextrous	69 (2.6%)
First language	English	2588 (96.2%)
	Other	102 (3.8%)
Ethnicity	White	2581 (95.9%)
	Unknown	10 (0.4%)
	Mixed or multiple ethnic groups	41 (1.5%)
	Asian or Asian British	34 (1.3%)
	Black, Black British, Caribbean or African	24 (0.9%)
Residence	United Kingdom	2671 (99.3%)
	Abroad	19 (0.7%)
Education	preGCSE	63 (2.3%)
	High School	1269 (47.2%)
	University Degree	1291 (48%)
	PhD	67 (2.5%)
Occupation	Worker	1089 (40.5%)
	Retired	916 (34.1%)
	Disabled / Not applicable / Sheltered employment	502 (18.7%)
	Homemaker	108 (4%)
	Unemployed / Looking for work	41 (1.5%)
	Student	21 (0.8%)
	Unknown	13 (0.5%)
Disease subtype	Benign	70 (2.6%)
	Relapsing-remitting	1451 (53.9%)
	Transitioning to secondary progressive	70 (2.6%)
	Secondary progressive	645 (24%)
	Primary progressive	351 (13%)
	Do not know	103 (3.8%)
Disease duration (min-max, mean)		0-63, 18 (missing = 1)

Table S5. Analysis of engagement with Stages 1 and 2. Demographics, disease subtype, disease duration, and disease severity across: i) participants from Stage 1 or Stage 2 who completed, in one or more sessions, vs who did not complete; ii) participants from Stage 1 who did vs did not return to take part in Stage 2; iii) participants from Stage 1 vs overall UK MS Register population; iv) overall participants from Stage 1 vs Stage 2; v) overall participants from Stage 1 vs independent sample from Stage 2. P-values were obtained using two-tailed t-tests, ANOVA, or chi-squared tests where appropriate. Significant p-values ($p \leq 0.05$) are highlighted in bold.

Groups	N	Age (min-max, mean)	Sex (%)	Disease subtype (%)	Disease duration (min-max, mean)	Disease severity (mean MSIS-29 motor norm / webEDSS)
Stage 1						
Completed in one session	2247 (73.3%)	16-87, 54.4 (missing = 2)	Female = 79<br>Male = 20.8<br>Other = 0.2<br>(missing = 2)	BN = 2.4<br>RR = 54.9<br>SP = 29.2<br>PP = 13.5<br>(missing = 512)	1-67, 20.3 (missing = 512)	40.5 / 4.8 (missing = 358 / 1320)
Completed in more than one session	158 (5.1%)	25-79, 54.9	Female = 82.9<br>Male = 17.1<br>Other = 0	BN = 1.6<br>RR = 50.4<br>SP = 32.3<br>PP = 15.7<br>(missing = 31)	0-46, 21.3 (missing = 31)	44.2 / 5.4 (missing = 24 / 96)
Did not complete	661 (21.6%)	20-82, 54.6 (missing = 2)	Female = 70.7<br>Male = 29<br>Other = 0.3<br>(missing = 2)	BN = 2<br>RR = 50.6<br>SP = 32.3<br>PP = 15.1<br>(missing = 159)	1-55, 20.9 (missing = 159)	45.7 / 5 (missing = 163 / 431)
P-values from group comparison	-	0.8225	**0.0001**	0.6045	0.4679	**0.0004 / 0.0282**
Stage 1 – returning	1267 (41.3%)	16-87, 56.5	Female = 77.7<br>Male = 22<br>Other = 0.2	BN = 2.4<br>RR = 49.4<br>SP = 32.3<br>PP = 15.9<br>(missing = 239)	1-64, 21 (missing = 239)	42 / 4.9 (missing = 103 / 669)
Stage 1 – non-returning	1799 (58.7%)	20-85, 52.9 (missing = 4)	Female = 77.2<br>Male = 22.6<br>Other = 0.2<br>(missing = 4)	BN = 2.1<br>RR = 57<br>SP = 28.3<br>PP = 12.6<br>(missing = 463)	0-67, 20.1 (missing = 463)	41.5 / 4.8 (missing = 442 / 1178)
P-values from group comparison	-	**7.0126e-17**	0.9242	**0.0027**	0.0606	0.6216 / 0.3225
Stage 1 – overall	3066	16-87, 54.4 (missing = 4)	Female = 77.4<br>Male = 22.4<br>Other = 0.2<br>(missing = 4)	BN = 2.2<br>RR = 53.7<br>SP = 30<br>PP = 14<br>(missing = 702)	0-67, 20.5 (missing = 702)	41.7 / 4.8 (missing = 545 / 1847)
Overall UK MS Register population (up to Spring 2022)	19188	18-90, 54.3	Female = 74.2<br>Male = 25.8	-	0-73, 20.1	-
P-values from group comparison	-	0.5196	**1.2366e-13**	-	0.1546	-
Stage 2						

Completed in one session	1897 (70.4%)	18-88, 53.8<br>(missing = 3)	Female = 78.8<br>Male = 21.1<br>Other = 0.1<br>(missing = 3)	BN = 2.8<br>RR = 56.1<br>TrnSP = 2.6<br>SP = 22.5<br>PP = 12.1<br>DK = 3.9<br>(missing = 3)	0-63, 17.8<br>(missing = 4)	43.2 / 4.9<br>(missing = 635 / 867)
Completed in more than one session	300 (11.1%)	21-86, 55.2	Female = 78.3<br>Male = 21.7<br>Other = 0	BN = 1.7<br>RR = 45.7<br>TrnSP = 2.7<br>SP = 26.7<br>PP = 18.7<br>DK = 4.7	0-60, 18.9	49.9 / 5.2<br>(missing = 99 / 126)
Did not complete	499 (18.5%)	20-80, 53.7<br>(missing = 2)	Female = 74.8<br>Male = 25.2<br>Other = 0<br>(missing = 2)	BN = 2.4<br>RR = 50.7<br>TrnSP = 2.6<br>SP = 28<br>PP = 13.1<br>DK = 3.2<br>(missing = 2)	0-58, 18.4<br>(missing = 2)	47.4 / 5.0<br>(missing = 207 / 270)
P-values from group comparison	-	0.1546	**0.0412**	**0.0099**	0.2448	**0.0007** / 0.0985
Stage 1 compared to Stage 2						
Stage 1 – overall	3066	16-87, 54.4<br>(missing = 4)	Female = 77.4<br>Male = 22.4<br>Other = 0.2<br>(missing = 4)	BN = 2.2<br>RR = 53.7<br>SP = 30<br>PP = 14<br>(missing = 702)	0-67, 20.5<br>(missing = 702)	41.7 / 4.8<br>(missing = 545 / 1847)
Stage 2 – overall	2696	18-88, 53.9<br>(missing = 5)	Female = 78<br>Male = 21.9<br>Other = 0.1<br>(missing = 5)	BN = 2.6<br>RR = 54<br>TrnSP = 2.6<br>SP = 24<br>PP = 13<br>DK = 3.8<br>(missing = 5)	0-63, 18<br>(missing = 6)	44.6 / 4.9<br>(missing = 941 / 1263)
P-values from group comparison	-	0.1184	0.3004	0.1718	**4.9524e-13**	**0.0005** / 0.2121
Stage 1 – overall	3066	16-87, 54.4<br>(missing = 4)	Female = 77.4<br>Male = 22.4<br>Other = 0.2<br>(missing = 4)	BN = 2.2<br>RR = 53.7<br>SP = 30<br>PP = 14<br>(missing = 702)	0-67, 20.5<br>(missing = 702)	41.7 / 4.8<br>(missing = 545 / 1847)
Stage 2 – independent	1429 (53%)	18-86, 50.9<br>(missing = 3)	Female = 78.1<br>Male = 21.8<br>Other = 0.1<br>(missing = 3)	BN = 2<br>RR = 58.8<br>TrnSP = 2.4<br>SP = 20.1<br>PP = 11.7<br>DK = 5<br>(missing = 3)	0-62, 16.1<br>(missing = 3)	45.3 / 4.9<br>(missing = 727 / 871)
P-values from group comparison	-	**3.6851e-20**	0.4552	**<1.8383e-05**	**2.6878e-27**	**0.0017** / 0.6876

Table S6. Eigenvalues and explained variance from factor analysis. Eigenvalues resulting from factor analysis of primary performance metrics across tasks from Stage 1 data (N by task in Table S1). The table reports the variance, proportional variance, and cumulative variance explained by each of the 6 selected factors, both before and after applying varimax rotation.

Unrotated Factors	1	2	3	4	5	6
Eigenvalue	4.06474402	1.87636668	1.23542992	1.14378247	1.08399374	1.0136389
Variance	3.393818	1.217887	0.580832	0.443161	0.393024	0.274042
Proportional variance	0.154264	0.055358	0.026401	0.020144	0.017865	0.012456
Cumulative variance	0.154264	0.209623	0.236024	0.256168	0.274033	0.286489
Rotated Factors	Information processing speed	Problem solving	Working Memory	Verbal abilities	Memory	Attention
Eigenvalue	4.06474402	1.87636668	1.23542992	1.14378247	1.08399374	1.0136389
Variance	1.867261	1.409980	0.994726	0.812941	0.797591	0.420265
Proportional variance	0.084875	0.064090	0.045215	0.036952	0.036254	0.019103
Cumulative variance	0.084875	0.148965	0.194180	0.231132	0.267386	0.286489

Table S7. People with Multiple Sclerosis (MS) sociodemographic characteristics Stage 3. Sociodemographic characteristics of people with MS from the Hammersmith Hospital who consented to be part of the UK MS Register and participated in Stage 3.

Sociodemographic characteristics		Participants
Total N		31
Age (min-max, mean)		28-76, 46.5
Sex	Female	23 (74.2%)
	Male	8 (25.8%)
	Other	0
Dominant hand	Right	29 (93.5%)
	Left	2 (6.5%)
	Ambidextrous	0
First language	English	23 (74.2%)
	Other	8 (25.8%)
Ethnicity	White	19 (61.3%)
	Mixed or multiple ethnic groups	2 (6.5%)
	Asian or Asian British	3 (9.7%)
	Black, Black British, Caribbean or African	4 (12.9%)
	Other ethnic group	3 (9.7%)
Residence	United Kingdom	31 (100%)
Education	preGCSE	1 (3.2%)
	High School	6 (19.4%)
	University Degree	23 (74.2%)
	PhD	1 (3.2%)
Occupation	Worker	18 (58.1%)
	Retired	4 (12.9%)
	Disabled / Not applicable / Sheltered employment	5 (16.1%)
	Homemaker	1 (3.2%)
	Unemployed / Looking for work	2 (6.5%)
	Student	1 (3.2%)
Disease subtype	Relapsing-remitting	27 (87.1%)
	Transitioning to secondary progressive	1 (3.2%)
	Secondary progressive	2 (6.5%)
	Primary progressive	1 (3.2%)
Disease duration (min-max, mean)		2-42, 16.2

Table S8. Correlations with Nine-Hole Peg Test. Two-tailed Pearson correlations between primary performance metrics and Nine-Hole Peg Test scores from the less impaired hand across participants, evaluated on Stage 3 data (N=31). P-values are reported to indicate the statistical significance of the observed correlations. Significant correlations ($p \leq 0.05$) are highlighted in bold.

Task	Correlation	P-value
Blocks	-0.0582	0.7557
Card Pairs	-0.2586	0.1602
Manipulations 2D	**-0.3664**	**0.0427**
Motor Control	**0.5749**	**0.0007**
Objects Memory Delayed	-0.3305	0.0694
Objects Memory Immediate	-0.3109	0.0887
SRT	0.3	0.1011
Switching Stroop	-0.0174	0.9259
Trail Making	**0.4037**	**0.0243**
Verbal Analogies	0.0458	0.8069
Word Definitions	-0.0403	0.8296
Words Memory Delayed	-0.2765	0.1321
Words Memory Immediate	-0.2437	0.1865

Table S9. Characterisation of people with Multiple Sclerosis (pwMS) with and without cognitive impairment. Prevalence, demographics, disease subtype, and disease duration in pwMS classified as having or not cognitive impairment, according to three criteria from the literature of varying stringency. Criteria were based on a combination of different abnormality cut-offs and critical numbers of abnormal parameters. P-values were obtained using two-tailed t-tests or chi-squared tests, as appropriate. Statistically significant p-values ($p \leq 0.05$) are highlighted in bold.

Criterion	Groups	Prevalence	Age (min-max, mean)	Sex (%)	Disease subtype (%)	Disease duration (min-max, mean)
Liberal<br>Abnormality threshold = 1SD<br>Critical number of abnormal parameters >=13%	Impaired	72.1%	18-85, 54.7	Female = 77.8<br>Male = 22.1<br>Other = 0.1	BN = 3<br>RR = 51.8<br>TrnSP = 2.2<br>SP = 25.4<br>PP = 13.5<br>DK = 4	0-63, 18.7
	Non-impaired	27.9%	22-84, 51.3	Female = 84.1<br>Male = 15.7<br>Other = 0.2	BN = 2.8<br>RR = 68.6<br>TrnSP = 2.8<br>SP = 13.3<br>PP = 9.3<br>DK = 3.2	0-50, 15.3
P-values from group comparison			**3.3179e-08**	**0.0084**	**2.8683e-09**	**2.8332e-08**
Fair<br>Liberal<br>Abnormality threshold = 1.5SD<br>Critical number of abnormal parameters >=20%	Impaired	29.4%	19-85, 55.7	Female = 71.7<br>Male = 28.3<br>Other = 0	BN = 1.9<br>RR = 43.4<br>TrnSP = 2.5<br>SP = 33.4<br>PP = 14.2<br>DK = 4.7	0-63, 20.2
	Non-impaired	70.6%	18-84, 52.9	Female = 82.9<br>Male = 17<br>Other = 0.2	BN = 3.4<br>RR = 61.9<br>TrnSP = 2.4<br>SP = 17.3<br>PP = 11.6<br>DK = 3.4	0-61, 16.7
P-values from group comparison			**2.8001e-06**	**2.6345e-07**	**2.0517e-14**	**3.8280e-09**
Conservative<br>Abnormality threshold = 2SD<br>Critical number of abnormal parameters >=20%	Impaired	14.6%	19-85, 56.7	Female = 70.7<br>Male = 29.3<br>Other = 0	BN = 1.5<br>RR = 37.3<br>TrnSP = 2.3<br>SP = 39.5<br>PP = 13.3<br>DK = 6.1	0-62, 22
	Non-impaired	85.4%	18-84, 53.2	Female = 81.1<br>Male = 18.8<br>Other = 0.1	BN = 3.2<br>RR = 59.8<br>TrnSP = 2.4<br>SP = 19<br>PP = 12.2<br>DK = 3.4	0-63, 17
P-values from group comparison			**1.2301e-05**	**0.0004**	**1.1694e-13**	**1.4607e-10**

Table S10. Early-stage data availability. Number of participants between 0 and 5 years post disease onset who completed each task at Stage 2 after preprocessing procedures were applied.

Task	N participants between 0 and 5 years post onset
Blocks	273
Card Pairs	278
Manipulations 2D	286
Motor Control	290
Objects Memory Delayed	224
Objects Memory Immediate	297
SRT	261
Switching Stroop	274
Trail Making	261
Verbal Analogies	265
Word Definitions	265
Words Memory Delayed	227
Words Memory Immediate	293

Table S11. Clusters characterisation. Demographics, education level, disease subtype, disease duration, and age at onset for people with Multiple Sclerosis (MS) across clusters. P-values were obtained using ANOVA or chi-squared tests, as appropriate. Statistically significant p-values (p ≤ 0.05) are highlighted in bold.

	Minimal Motor + Moderate Cognitive	Severe Motor + Mild Cognitive	Minimal Motor + No Cognitive	Severe Motor + Severe Cognitive	P-values from clusters comparison
Count	307	403	301	169	-
Age (min-max, mean)	19-80, 52.5	25-84, 59.2	25-81, 53.9	31-85, 56.8	**1.7832e-16**
Sex (%)	Female = 82.4<br>Male = 17.6	Female = 77.9<br>Male = 22.1	Female = 81.4<br>Male = 18.3<br>Other = 0.3	Female = 64.5<br>Male = 35.5	**0.0002**
Education (%)	preGCSE = 1.3<br>High School = 38.4<br>University = 56.4<br>PhD = 3.9	preGCSE = 3.2<br>High School = 51.9<br>University = 43.2<br>PhD = 1.7	preGCSE = 1.3<br>High School = 35.9<br>University = 57.5<br>PhD = 5.3	preGCSE = 3.6<br>High School = 56.8<br>University = 38.5<br>PhD = 1.2	**3.7982e-07**
Subtype (%)	BN = 5.2<br>RR = 75.6<br>TrnSP = 1.3<br>SP = 8.1<br>PP = 6.2<br>DK = 3.6	BN = 1<br>RR = 27.8<br>TrnSP = 4<br>SP = 38.7<br>PP = 26.6<br>DK = 2	BN = 4<br>RR = 72.8<br>TrnSP = 2<br>SP = 9.6<br>PP = 7.3<br>DK = 4.3	BN = 1.2<br>RR = 32.5<br>TrnSP = 2.4<br>SP = 40.2<br>PP = 19.5<br>DK = 4.1	**1.9387e-58**
Disease duration (min-max, mean)	0-49, 16	1-63, 21.3	0-61, 16.2	1-62, 21.1	**5.8240e-13**
Age at onset (min-max, mean)	11-73, 36.5	1-70, 37.9	11-72, 37.8	7-68, 35.7	0.1005

Table S12. Questionnaire transcript. Specific questionnaire items analysed in this study. The same sociodemographic questions were asked in the Great British Intelligence Test (GBIT) study and were analysed for the controls used in this study to derive regression-based norms. For each item, the categories that were derived and used for analysis are also reported.

Question	Possible answers	Derived categories
How old are you?	Drop-down list with numbers from 0 to 100+	1. 16-20<br>2. 21-30<br>3. 31-40<br>4. 41-50<br>5. 51-60<br>6. 61-70<br>7. 71-80<br>8. 81-90
Sex	1. Male<br>2. Female<br>3. Other	1. Male<br>2. Female<br>3. Other
Are you left or right handed?	1. Right handed<br>2. Left handed<br>3. Ambidextrous	1. Right handed<br>2. Left handed<br>3. Ambidextrous
First Language	Drop-down list with all possible languages	1. English<br>2. Other
To ensure we have a representative sample of the population, please indicate your ethnicity?	Stage 1<br>1. White European or North American<br>2. Sub-saharan African or Afro-american<br>3. North African<br>4. West-central Asian<br>5. East Asian<br>6. Indian, South Asian or South-East Asian<br>7. Rom, Sinti or Bedouin<br>8. American Hispanic<br>9. Mixed ethnicity<br>10. Unknown<br><br>Stages 2 and 3<br>1. White English/ Welsh/Scottish/ Northern Irish/British<br>2. White Irish<br>3. White Gypsy or Irish Traveller<br>4. Other White<br>5. White and Black Caribbean<br>6. White and Black African<br>7. White and Asian<br>8. Other Mixed/ Multiple ethnic group<br>9. Indian<br>10. Pakistani<br>11. Bangladeshi<br>12. Chinese<br>13. Other Asian<br>14. African<br>15. Caribbean<br>16. Other Black<br>17. Arab<br>18. Other	1. White<br>2. Asian or Asian British<br>3. Black, Black British, Caribbean or African<br>4. American Hispanic<br>5. Mixed or multiple ethnic groups<br>6. Other ethnic group<br>7. Unknown
Country of residence	Drop-down list with all possible countries	1. United Kingdom<br>2. Abroad
What is your level of education	Stage 1<br>1. No schooling<br>2. Primary/ Elementary school<br>3. Secondary school/ High school diploma<br>4. University degree<br>5. PhD<br><br>Stages 2 and 3<br>1. No schooling<br>2. Primary/ Elementary school<br>3. High School – GCSEs<br>4. High School – A-Levels<br>5. University degree<br>6. PhD	1. preGCSE<br>2. High School<br>3. University Degree<br>4. PhD
What is your occupational status?	1. Worker<br>2. Student<br>3. Homemaker<br>4. Retired<br>5. Unemployed/Looking for work<br>6. Disabled/Not applicable/Sheltered employment<br>7. Unknown	1. Worker<br>2. Student<br>3. Homemaker<br>4. Retired<br>5. Unemployed/Looking for work<br>6. Disabled/Not applicable/ Sheltered employment<br>7. Unknown

Stages 2 and 3 only		
What is your Multiple Sclerosis subtype at the moment?	1. Benign<br>2. Relapsing Remitting<br>3. Primary Progressive<br>4. Secondary Progressive<br>5. Transitioning to Secondary Progressive<br>6. Do not know	1. Benign<br>2. Relapsing Remitting<br>3. Primary Progressive<br>4. Secondary Progressive<br>5. Transitioning to Secondary Progressive<br>6. Do not know
What year did you first have symptoms?	Drop-down list with years from 1922 to 2024	Disease duration<br>1. 0-9<br>2. 10-19<br>3. 20-29<br>4. 30-39<br>5. >=40

Table S13. Cognitron tasks design. Summary of the Cognitron tasks used in the study, detailing task descriptions, derived performance scores, targeted cognitive domains, and the stage/s of the study in which they were employed.

Task	Description	Performance scores	Measured domain/s	Used in stage/s
Words Memory Immediate	The participant is presented with a string of 12 words. They are then presented with another set of 24 words that may or may not have been in the original string. They must identify which words in this new set were in the original string. This task is performed at the beginning of the battery and is paired with a follow up task at the end of the battery.	Accuracy = percent correct answers<br>Response Time = median RT across trials with correct answers	Short-term recognition memory (language)	1, 2, 3
Words Memory Delayed	This task is paired with the Words Memory Immediate task. The participant is presented with a set of 24 words that may or may not have been in the Words Memory Immediate task at the beginning of the battery. They must identify which words in this new set were in the original string and which were not. The difference is in the delay between word presentation and word recall.	Accuracy = percent correct answers<br>Response Time = median RT across trials with correct answers<br>Elements Forgotten = percent of items that have been forgotten	Medium-term recognition memory (language)	1, 2, 3
Objects Memory Immediate	The participant is presented with a string of 20 object silhouettes that they must remember. They are then presented with a string of grids each containing one of the original objects that were displayed previously. Along with the target object, there are also 7 other distractor objects that range in similarity to the target object over three parameters (orientation, pose and item). The participant must select the target object from the distractor objects, for each grid. This task is performed at the beginning of the battery and is paired with a follow up task at the end of the battery.	Accuracy = percent correct answers<br>Response Time = median RT across trials	Short-term recognition memory (objects)	2, 3
Objects Memory Delayed	This task is paired with the Objects Memory Immediate task. The participant is presented again with the string of grids without being reminded of the original string of target objects. They must again pick out the target objects from the grids. The difference is in the delay between object presentation and object recall.	Accuracy = percent correct answers<br>Response Time = median RT across trials<br>Elements Forgotten = percent of items that have been forgotten	Medium-term recognition memory (objects)	2, 3
Card Pairs	This is a variant of the classic Card Pairs game. The participant is shown a set of 6 pairs of cards. After a set time, the cards are turned over and the participant must select cards and match them with their corresponding pair. This is repeated for 5 trials.	Accuracy = percent correct answers<br>Response Time = mean time to complete a set	Associative working memory	1, 2, 3

Digit Span	The participant is presented with a string of digits that they must recall. After the participant correctly remembers a string of digits, the next string increases in length by one. The game ends when the participant answers incorrectly 3 times for a given string length.	Accuracy = maximum number of digits remembered<br>Response Time = median recall time across elements correctly remembered	Working memory	1
Spatial Span	The participant is presented with a string of locations on a grid that they must recall. After the participant correctly remembers a string of positions, the next string increases in length by one. The game ends when the participant answers incorrectly 3 times for a given string length.	Accuracy = maximum number of locations remembered<br>Response Time = median recall time across elements correctly remembered	Working memory	1
Pair Associative Learning (PAL)	The participant is presented with a grid in which a series of images appears one by one in different grid squares. The participant is then presented with each image they have previously seen, and they must indicate which grid square it appeared in. After the participant correctly remembers a series of images, the next series increases in length by one. The game ends when the participant answers incorrectly 3 times for a given series length.	Accuracy = maximum number of images remembered<br>Response Time = median recall time across elements correctly remembered	Associative working memory	1
Motor Control	The participant is presented with a string of small targets that appear all over the screen. They must click on the targets as quickly and accurately as possible.	Accuracy = -mean distance from target centre<br>Response Time = mean RT across trials	Reaction time	1, 2, 3
Simple Reaction Task (SRT)	The participant is presented with a string of targets. They must click anywhere on the screen as soon as the target appears.	Accuracy = percent correct answers<br>Response Time = median RT across trials	Reaction time	1, 2, 3
Complex Reaction Task (CRT)	The participant is presented with a string of arrows pointing left or right. They must click on the side of the screen indicated by the arrow as quickly as possible.	Accuracy = percent correct answers<br>Response Time = median RT across trials<br>Switch Cost Accuracy = change in percent correct answers across trials for which the side to click on changes compared to previous trial vs stays the same<br>Switch Cost Response Time = change in median RT across trials for which the side to click on changes compared to previous trial vs stays the same<br>CRT – SRT (Choice Cost) = change of median RT across trials from SRT to CRT (when there is a choice involved)	Reaction time	1
Trail Making	The participant is presented with a set of 26 numbered tiles, which they must click in order from 1 upwards. They are then presented with a set of 26 tiles with numbers and letters. They must alternate between clicking on the numbers and letters, but in both cases	Accuracy = percent correct answers<br>Response Time = mean time to complete a set<br>Switch Cost Response Time = difference in RT between completing a set with and without having to switch from clicking numbers to letter	Cognitive flexibility, task switching	1, 2, 3

	clicking in ascending order (e.g., 1-A-2-B-3-C-4-D-5-E… up to 13-M).			
Switching Stroop	The participant is presented with a coloured block (either red or blue) and on either side the words "red" and "blue". These words are drawn with either red or blue ink. At the top of the task, there is a condition which reads either "ink" or "text". If the condition reads "ink" then the participant must click on the word that has the same ink colour as the square in the middle. If the condition reads "text" then the participant must click on the word that describes the colour of the square in the middle. The condition switches pseudo-randomly as the task progresses and so do the words on either side of the block and their ink. The task includes 60 trials.	Accuracy = percent correct answers<br>Response Time = median RT across trials<br>Switch Cost Accuracy = change in percent correct answers across trials for which the condition changes compared to previous trial vs remains the same<br>Switch Cost Response Time = change in median RT across trials for which the condition changes compared to previous trial vs remains the same<br>Incongruency Cost Accuracy = change in percent correct answers across trials for which text and ink of the stimuli are incongruent vs congruent<br>Incongruency Cost Response Time = change in median RT across trials for which text and ink of the stimuli are incongruent vs congruent	Cognitive control, task switching	1, 2, 3
Blocks	The participant is presented with a set of coloured blocks in a grid and a target configuration made of a subset of the blocks. The participant must remove coloured blocks by clicking on them to match the target configuration. Blocks fall with gravity. The task includes 15 trials.	Accuracy = percent correct answers<br>Response Time = median RT across trials	2D spatial planning	1, 2, 3
Tower of London (TOL)	This is a variant of the classic Tower of London task. The participant is presented with 2 sets of 3 pegs with coloured rings on them. They must work out the minimum number of moves it would take to get from one configuration of rings to the other. The task includes 10 trials.	Accuracy = percent correct answers<br>Response Time = median RT across trials	2D spatial planning	1
Manipulations 2D	The participant is presented with a target grid of coloured squares and 4 probe grids. One of the probe grids is the same as the target grid but has been rotated 90, 180 or 270 degrees; the other probes have been subtly changed from the target grid. The participant must identify the probe that is the same configuration as the target. The participants have 3 minutes (Stage 1) or 2 minutes (Stage 2 and 3) to complete as many trials as possible.	Accuracy = number of correct answers<br>Response Time = median RT across trials	2D spatial reasoning	1, 2, 3
Picture Completion	The participant is presented with a set of 12 images that have pieces missing from them, and a set of pieces on the side of each image. The pieces may have been rotated from their original orientation. The participant must select which of the missing pieces goes in which of the empty slots in the image. In some of the trials there are also distractor pieces.	Accuracy = percent correct answers<br>Response Time = total time taken<br>Distractor Cost Accuracy = change in percent correct answers across trials with or without distractors<br>Distractor Cost Response Time = change in total time taken to complete trials with or without distractors	2D spatial reasoning	1

Four/Faulty Towers	The participant is given a set of four 3D perspective images with buildings and trees. Three of the images are the same but rotated at 90, 180 or 270 degrees; one is different. The participant must select which image is different. The task includes 12 trials.	Accuracy = percent correct answers<br>Response Time = median RT across trials	3D spatial reasoning	1
Target Detection	The participant is presented with an abstract shape as a target. They are then presented with a constantly changing set of different abstract shapes. They must quickly click on the target shape every time it appears while ignoring the distractor shapes. The grid refreshes 120 times and each shape stays on the grid for 4 refreshes.	Accuracy = percent targets detected<br>Response Time = median RT across targets detected	Attention	1
Spotter	The participant is presented with a string of grey static grids that flash quickly onto the screen and then off again. Some of the grids contain numbers with varying degrees of decipherability. The participant must pay close attention to the grids and click the screen as quickly as possible every time they see a "0" in one of the grids. The task is long and tests the participants' ability to maintain focus; it lasts 4 minutes (in Stage 1) or 5 minutes (in Stage 2 and 3). In Stages 2 and 3 a calibration block was added to adjust number decipherability.	Accuracy = percent zeros detected<br>Response Time = mean RT across trials in which zeros were correctly detected	Sustained attention	1, 2
Verbal Analogies	The participant is presented with a set of analogies and must decide if each analogy is true or false. For example, a true analogy would be "Apple is to fruit as hammer is to tool", whereas an incorrect analogy would be "Apple is to fruit as large is to small". The participants have 3 minutes (Stage 1) or 2.5 minutes (Stage 2 and 3) to complete as many trials as possible. The analogies are sampled from a problem space of 120 analogies (Stage 1) or 180 analogies (Stage 2 and 3).	Accuracy = number of correct answers<br>Response Time = median RT across trials	Verbal reasoning	1, 2, 3
Word Definitions	The participant is given a set of 21 English words with varying rarity; they must select the correct definition from a choice of 4. They have 20 seconds to answer for each word.	Accuracy = percent correct answers<br>Response Time = median RT across trials	Crystallised intelligence	1, 2, 3
Bees	The participant is presented with a set of "hives". Each hive contains two sets of bees with different colours. The participant must guess which of the sets of bees is most numerous by uncovering one bee at a time at random. The more bees they uncover the more certain they can be, but the longer it will take. The task included 15 trials.	Accuracy = percent correct answers<br>Response Time = mean RT across samples (how long they think about sampling another bee)<br>Mean Samples = mean number of bees sampled before deciding<br>Mean Difference = mean difference between number of samples of each type before deciding	Impulsivity	1

Emotion Discrimination	The participant is presented with a string of pairs of faces expressing emotions. They must discern whether the emotions on the faces are the same or different to each other. The task includes 50 trials.	Accuracy = percent correct answers<br>Response Time = median RT across trials<br>Incongruency Cost Accuracy = change in percent correct answers when comparing faces showing different vs similar emotions<br>Incongruency Cost Response Time = change in median RT when comparing faces showing different vs similar emotions	Emotional intelligence	1

Table S14. Control data availability. Number of controls available for each task in its original version. For tasks where normative data from the modified version were used in Stages 2 and 3, the number of controls available for the modified version is also reported.

Task	N controls original version	N controls modified version
Bees	4203	-
Blocks	389575	-
Card Pairs	6204	-
CRT	5391	-
Digit Span	387739	-
Emotion Discrimination	387704	-
Four Towers	102895	-
Manipulations 2D	388723	-
Motor Control	25932	-
Objects Memory Delayed	-	17240
Objects Memory Immediate	-	21144
PAL	3375	-
Picture Completion	2307	-
Spatial Span	388979	-
Spotter	3998	-
SRT	5620	-
Switching Stroop	6228	-
Target Detection	387746	-
TOL	379549	-
Trail Making	7160	-
Verbal Analogies	387559	19431
Word Definitions	388653	-
Words Memory Delayed	93300	-
Words Memory Immediate	99649	-

Table S15. Sociodemographic characteristics of controls. Sociodemographic characteristics of all participants from the Great British Intelligence Test (GBIT) study who were retained as controls for the present study.

Sociodemographic characteristics		Participants
Total N		389,925
Age (min-max, mean)		16-81, 44.9
Sex	Female	178,875 (45.9%)
	Male	207,366 (53.2%)
	Other	3,684 (0.9%)
Dominant hand	Right	336,841 (86.4%)
	Left	42,550 (10.9%)
	Ambidextrous	10,534 (2.7%)
First language	English	350,658 (89.9%)
	Other	39,267 (10.1%)
Ethnicity	White	331,039 (84.9%)
	Unknown	5,213 (1.3%)
	Mixed or multiple ethnic groups	9,979 (2.6%)
	Asian or Asian British	31,077 (8%)
	Black, Black British, Caribbean or African	3,553 (0.9%)
	American Hispanic	9,023 (2.3%)
	Other ethnic group	41 (0.01%)
Residence	United Kingdom	339,824 (87.2%)
	Abroad	50,101 (12.8%)
Education	preGCSE	10,729 (2.8%)
	High School	129,806 (33.3%)
	University Degree	230,251 (59.1%)
	PhD	19,139 (4.9%)
Occupation	Worker	266,757 (68.4%)
	Retired	56,425 (14.5%)
	Disabled / Not applicable / Sheltered employment	3,998 (1%)
	Homemaker	12,139 (3.1%)
	Unemployed / Looking for work	11,921 (3.1%)
	Student	36,241 (9.3%)
	Unknown	2,444 (0.6%)

Table S16. Standard in-person neuropsychological assessment. Summary of neuropsychological tests administered in Stage 3 as part of the standard in-person battery. For each are reported a description of how the test was carried out, the scoring system, what normative data were used for norming, what sociodemographic factors were accounted for in norming, and whether the test is included in the MACFIMS battery. Analogous information is reported for the Nine-Hole Peg Test.

Test	Test description	Test scoring	Normative data source	Factors accounted for	Is in MACFIMS?
California Verbal Learning Test – Second Edition (CVLT-II) Immediate Recall	The examiner reads out a list of 16 words to the participant. The participant is then asked to recall as many words from the list as they can. The test consists of 5 trials, and the string of words is repeated by the examiner at the start of each trial. This test is the first in the battery and is coupled with the CVLT-II Delayed Recall which is the second to last in the battery.	1 point is awarded for each word recalled. The final score is the total number of words recalled across all five learning trials (maximum of 80 points).	Parmenter BA, Testa SM, Schretlen DJ, Weinstock-Guttman B, Benedict RH. The utility of regression-based norms in interpreting the minimal assessment of cognitive function in multiple sclerosis (MACFIMS). *J Int Neuropsychol Soc*. 2010;16(1):6-16. doi:10.1017/S1355617709990750	Age, age squared, sex, education	Yes
California Verbal Learning Test – Second Edition (CVLT-II) Delayed Recall	The examiner asks the participant to recall after a distraction phase as many words as possible from the original list of 16 words learned during the CVLT-II Immediate Recall with no cues provided.	1 point is awarded for each word recalled (maximum of 16 points).	Parmenter BA, Testa SM, Schretlen DJ, Weinstock-Guttman B, Benedict RH. The utility of regression-based norms in interpreting the minimal assessment of cognitive function in multiple sclerosis (MACFIMS). *J Int Neuropsychol Soc*. 2010;16(1):6-16. doi:10.1017/S1355617709990750	Age, age squared, sex, education	Yes
Brief Visuospatial Memory Test – Revised (BVMT-R) Immediate Recall	The examiner presents the participant a sheet with a set of 6 figures drawn on it which they have 10 seconds to memorise. The examiner then hides the sheet and asks the participant to redraw as many figures as they remember. The test consists of 3 trials and the figures are shown to the participant for 10 seconds at the start of each trial. This task is the second task in the battery and is coupled with the BVMT-R Delayed Recall which is at the end of the battery.	A maximum of 2 points is awarded for each figure drawn: 1 point if the figure is drawn correctly and 1 point if the figure is drawn in the correct position on the sheet. The final score is the total number of points across all three learning trials (maximum of 36 points).	Parmenter BA, Testa SM, Schretlen DJ, Weinstock-Guttman B, Benedict RH. The utility of regression-based norms in interpreting the minimal assessment of cognitive function in multiple sclerosis (MACFIMS). *J Int Neuropsychol Soc*. 2010;16(1):6-16. doi:10.1017/S1355617709990750	Age, age squared, sex, education	Yes
Brief Visuospatial Memory Test	The examiner asks the participant to redraw as many figures as they can	A maximum of 2 points is awarded for each figure drawn: 1	Parmenter BA, Testa SM, Schretlen DJ, Weinstock-Guttman	Age, age squared, sex, education	Yes

– Revised (BVMT-R) Delayed Recall	from the original sheet shown during the BVMT-R Immediate Recall with no cues provided.	point if the figure is drawn correctly and 1 point if the figure is drawn in the correct position on the sheet (maximum of 12 points).	B, Benedict RH. The utility of regression-based norms in interpreting the minimal assessment of cognitive function in multiple sclerosis (MACFIMS). *J Int Neuropsychol Soc*. 2010;16(1):6-16. doi:10.1017/S1355617709990750		
Symbol Digit Modalities Test (SDMT)	The participant is presented with a grid of symbols. Each symbol is assigned a corresponding number in the grid. The participant is then also presented with a larger grid of the same symbols but without any corresponding numbers. The aim of the test is for the participant to go back and forth between the small and large grid as fast as they can and give the corresponding number to each symbol they read in the large grid. The participant has 90 seconds to get as far down the large grid as they can.	1 point is awarded for every correctly numbered symbol.	Parmenter BA, Testa SM, Schretlen DJ, Weinstock-Guttman B, Benedict RH. The utility of regression-based norms in interpreting the minimal assessment of cognitive function in multiple sclerosis (MACFIMS). *J Int Neuropsychol Soc*. 2010;16(1):6-16. doi:10.1017/S1355617709990750	Age, age squared, sex, education	Yes
Controlled Oral Word Association Test (COWAT)	This test consists of 3 trials. For the first trial, the participant has 60 seconds to name as many words as they can starting with the letter F. The second and third trials are the same but with the letters A and S respectively. No words with capital letters are allowed (no first names nor cities) and no word associations are allowed i.e. if the participant says “fire”, they can’t follow that with “fireman”.	1 point is awarded for each correct word generated. The final score is the total number of points across all three trials.	Parmenter BA, Testa SM, Schretlen DJ, Weinstock-Guttman B, Benedict RH. The utility of regression-based norms in interpreting the minimal assessment of cognitive function in multiple sclerosis (MACFIMS). *J Int Neuropsychol Soc*. 2010;16(1):6-16. doi:10.1017/S1355617709990750	Age, age squared, sex, education	Yes
Judgement of Line Orientation Test (JLO)	The participant is presented with a sheet of paper that is split into two halves. In the top half of the sheet are two angled lines and in the bottom half of the sheet is a semicircle with a set of 11 numbered lines separated 18 degrees from each other. The participant is asked to match the two angled lines to the corresponding numbered lines on the semicircle. The test consists of 30 items and	1 point is awarded every time the two angled lines are correctly matched (maximum of 30 points).	Parmenter BA, Testa SM, Schretlen DJ, Weinstock-Guttman B, Benedict RH. The utility of regression-based norms in interpreting the minimal assessment of cognitive function in multiple sclerosis (MACFIMS). *J Int Neuropsychol Soc*. 2010;16(1):6-16. doi:10.1017/S1355617709990750	Age, age squared, sex, education	Yes

	the angled lines are in a different position every time.				
Delis-Kaplan Executive Function System (D-KEFS) Sorting Test	The participant is presented with 6 cards (card set 1). Each card has a kind of colour, shape and size with a different word on it. The participant is asked to look at the cards and group them into two groups of three cards according to a common characteristic i.e. each group of three cards must have something in common which the participant must describe to the examiner. The participant must make as many groups as possible from a possible 8 groups that can be made (e.g. large cards vs small cards or red cards vs blue cards).	This task is scored according to two different criteria: sorting and description. For the sorting criteria, 1 point is awarded each time the cards are correctly grouped (maximum of 8 points). For the description criteria, 2 points are awarded each time a group is correctly described meaning a total of 4 points can be awarded for each sort if both groups are correctly described (maximum of 32 points).	Delis DC, Kaplan E, Kramer JH. Delis-Kaplan Executive Function System (D-KEFS) Examiner's Manual. San Antonio, TX: The Psychological Corporation; 2001.	Age	Yes
Victoria Stroop Test (Stroop)	This test consists of 3 trials. In the first trial (stroop dots), the participant is presented with a 4x6 grid of coloured dots and is asked to go through the grid as fast as they can whilst saying the colour of each dot. In the second trial (stroop words), the participant is presented with another 4x6 grid but with random coloured words and is asked once again to go through the grid as fast as they can whilst saying the colour of each word. In the final trial (stroop colours), the participant is presented with a final 4x6 grid of coloured words. However, this time the words are colours, and the participant is asked to go through the grid as fast as they can saying the colour of the word and not the word itself (which are always mismatched). For example, if the word is “Green” but written in the colour “Red”, the participant is required to say “Red”.	The raw score for each trial is the total number of seconds taken to complete the trial. Each trial is scored separately. The stroop interference score is calculated by dividing the stroop colours by the stroop dots raw scores.	Troyer AK, Leach L, Strauss E. Aging and response inhibition: Normative data for the Victoria Stroop Test. *Neuropsychol Dev Cogn B Aging Neuropsychol Cogn*. 2006;13(1):20-35. doi:10.1080/138255890968187	Age	No
Trail Making Test (TMT)	This test consists of two trials. For the first trial, the participant is presented with a set of numbered dots ranging from 1 to 25 and is asked to connect the dots in	Each trial is scored separately. The final scores are the total number of seconds it took the participant to complete the trials.	Karstens AJ, Christianson TJ, Lundt ES, et al. Mayo normative studies: regression-based normative data for	Age, age squared, sex, education	No

	ascending order starting from 1 and reaching 25. For the second trial, they are presented with a set of numbers and letters, and they must alternate between connecting numbers and letters, again in ascending order (e.g. 1-A-2-B-3-C-4-D-5-E…).		ages 30-91 years with a focus on the Boston Naming Test, Trail Making Test and Category Fluency. *J Int Neuropsychol Soc*. 2024;30(4):389-401. doi:10.1017/S1355617723000760		
Nine-Hole Peg Test (9HPT)	The participant is presented with a plastic board with 9 holes and a set of pegs. The participant is asked to place a peg in each hole as fast as they can and one by one, to complete the whole board, before removing each peg as fast as they can and one by one, to finish the trial. The test consists of four trials; two trials are performed with the participant's left hand and two trials are performed with the participant's right hand.	The score for each trial is the total number of seconds it took the participant to complete the trial. The scores for the left and right hand are averaged separately to generate one final score for each hand.	Erasmus LP, Sarno S, Albrecht H, Schwecht M, Pöllmann W, König N. Measurement of ataxic symptoms with a graphic tablet: standard values in controls and validity in Multiple Sclerosis patients. *J Neurosci Methods*. 2001;108(1):25-37. doi:10.1016/s0165-0270(01)00373-9	-	-

Supplementary Notes

Note S1: Factor analysis solution validation and stability

S1.1: Validity of factor structure

To assess the validity and the stability of the factor analysis solution, we implemented an iterative split-half cross-validation procedure as follows:

1. We randomly split the dataset into two subgroups, stratifying by the number of tasks completed per participant.
2. We performed factor analysis independently on each subgroup, retaining 6 factors and applying varimax rotation to the resulting loading matrices.
3. We compared the two resulting loading matrices by computing the average correlation between matched factors. For factor matching, we used the Hungarian algorithm, which globally optimised factor assignment to maximise the total correlation.
4. We repeated steps 1-3 over 100 iterations to obtain confidence intervals.
5. Correlations were averaged across iterations, and confidence intervals were calculated.

We observed an averaged matched-factor correlation of **0.74** (std=0.05) indicating a high degree of consistency across data splits. An example comparison of loading matrices is presented in Fig. S5. Loadings are sorted according to their matching order. The example matrices exhibit a correlation of **0.72**, with qualitatively similar loading patterns across the two subgroups.

S1.2: Stability of factor structure across sample sizes

To assess how factor structure stability varies with sample size—and to determine whether the solution was stable at the available sample size—we repeated the above split-half validation procedure while varying the subgroup sample size from 100 to 1524 (i.e., half of the full dataset), in increments of 20. For each sample size, we randomly selected two subgroups of the specified size, stratifying by the number of tasks completed per participant. We then performed factor analysis independently on each subgroup and matched the resulting factors using the Hungarian algorithm. This procedure was repeated 50 times per sample size, and the average matched-factor correlation across iterations was computed. Finally, we plotted the average matched-factor correlation as a function of sample size (Fig. S6). The resulting stability curve shows that the correlation is reaching a plateau at the maximum sample size tested, suggesting that the 6-factor structure is stable at the available sample size.

Residual variability in matched factors is likely attributable to the inherent noise in cognitive task performance and to the sparsity of the score matrix, which required the use of the pairwise deletion method to estimate the correlation matrix. Nonetheless, we considered an average matched-factor correlation of **0.74** to reflect sufficient reliability for our intended application—namely, to guide the selection of tasks that broadly capture cognitive domains while minimizing redundancy in the final battery.

Note S2: Clustering stability analysis

S2.1: Clustering stability analysis procedures

To choose the best clustering method given our dataset, we fixed the clustering configurations and performed clustering stability analysis in the following way:

1. For a range of sample sizes from 100 to 590 (i.e., half of our dataset size) in increments of 10, we repeatedly drew without replacement pairs of random subsamples of size n from the full dataset. Subsamples were allowed to overlap.
2. We applied the same clustering algorithm (with a fixed number of clusters k) independently to each subsample.
3. We matched clusters between the two resulting solutions using the Hungarian algorithm, aligning the clusters based on feature-level similarity (computed over clustering variables: either 13 cognitive and 2 motor features or the number of components retained from PCA).
4. We evaluated the similarity between the two clustering solutions using the following metrics:
    a. On individuals overlapping between the two subsamples only:
        i. Adjusted Rand Index (ARI) = compares pairs of samples and counts those assigned to the same or different clusters in both solutions: ARI=1 indicates perfect match, ARI=0 indicates random labelling, and ARI<0 indicates worse than random match.
        ii. Normalised Mutual Information (NMI) = quantifies the amount of shared information between two clustering solutions: NMI=1 for identical clustering and NMI=0 for completely independent solutions.
    b. On the full subsamples and across matched clusters:
        i. Accuracy similarity score = average difference in cluster-wise means across 10 accuracy-based cognitive features.
        ii. Response time similarity score = average difference in cluster-wise means across 3 RT-based cognitive features.
        iii. Patient-reported outcome similarity score = average difference in cluster-wise means across MSIS-29 motor and MSWS-12.
        iv. Cumulative similarity score = unweighted average of the accuracy, RT and PRO similarity scores (main similarity score summarising overall similarity in the three feature domains between matched clusters).
        v. Overall similarity score = average difference in cluster-wise means across all features (matching is based on minimising this score).
5. We repeated steps 2-4 for multiple iterations (M = 100) per sample size and aggregated the similarity scores across iterations resulting in one mean value with 95% confidence interval for each similarity score and sample size tested. This yielded a stability curve for each similarity score as a function of sample size.

We repeated this analysis under different configurations:

- clustering algorithms: Agglomerative Hierarchical Clustering (AHC) using different linkage methods (ward, average and complete) and K-means
- number of clusters: k = 3, 4, and 5
- dimensionality reduction: using principal component analysis (PCA) to reduce the number of clustering features before applying the chosen clustering algorithm and varying the number of components selected from 5 (the optimal number using the Kaiser's criterion) to 11 (the number of components that explain >90% of the total variance).

Finally, we plotted the stability curves and compared them across different solutions to see if each solution reached stability and identify which solution was the most stable and robust for our data given our sample size.

S2.2: Clustering stability analysis results

Fig. S10 presents the stability curves for different clustering algorithms and cluster numbers. Among the Agglomerative Hierarchical Clustering solutions, Ward's linkage is the method that consistently yielded the lowest cumulative and overall similarity scores across all tested cluster numbers. In contrast, average linkage produced the highest ARI and NMI, indicating higher agreement in cluster assignments between subsamples. However, all AHC solutions were outperformed by K-means clustering across all similarity metrics. K-means was therefore selected as the primary clustering algorithm. Stability curves for K-means with different cluster numbers were very similar, but the solution with 4 clusters demonstrated slightly better performance across multiple metrics. Consequently, the 4-cluster K-means solution was retained. Notably, the stability curves for K-means had not reached an asymptote, indicating that increased sample size could further enhance cluster stability. Given that our full dataset comprises double the size evaluated in the subsampling analysis, our final solution likely achieved improved stability.

We next investigated whether dimensionality reduction via PCA prior to clustering could further enhance stability. For completeness, this analysis was conducted for both AHC (restricted to the linkage method that performed better in the previous stability analysis, Ward's method) and K-means, fixing the number of clusters to 4. Fig. S11 shows the stability curves resulting from AHC and K-means with varying numbers of PCA components selected as input features for clustering. The number of components selected had limited impact on most stability metrics; however, for overall similarity, lower-dimensional representations performed slightly better, with five components yielding the most stable results.

Finally, Fig. S12 compares clustering stability with and without PCA (5 components selected) upstream for both algorithms. Applying PCA prior to clustering did not improve the clustering stability, hence why we decided not to use it.

In summary, we selected K-means with 4 clusters and 15 features as the final clustering algorithm. This configuration produced the most stable solution among those tested and is expected to benefit further from a larger sample size, as indicated by the fact that the corresponding stability curves had not yet plateaued when using half of the available dataset as sample size.

Note S3: Modifications in task designs between Stage 1 and Stage 2

S3.1: Task design modifications

The following modifications were applied to task design between Stage 1 and Stage 2:

1. For all the tasks, the orientation in which they were presented was changed from horizontal to vertical to ensure better compatibility and deployability via smartphones given recent updates to iOS.
2. For Verbal Analogies and Manipulations 2D, which are both timed tasks, task duration was reduced respectively from 3 to 2.5 minutes and from 3 to 2 minutes.
3. For Verbal Analogies, the problem space from which the presented analogies are sampled was increased from 120 to 180.
4. For Spotter, a calibration block was added in the beginning to adjust numbers decipherability, no consecutive targets were allowed, and task duration was increased from 4 to 5 minutes.

S3.2: Impact of task modifications on performance

To evaluate the impact of task modifications on performance, data from Stage 1, collected utilising original task versions, were compared with data from Stage 2, collected utilising modified task versions. For Stage 1, all users were included. For Stage 2, only users performing each task for the first time were included, to avoid capturing learning effects across stages. The datasets from both stages were combined, and a label was added to each observation to indicate whether it was generated from a task in its original version (Stage 1) or in its modified version (Stage 2). The analysis was conducted on raw performance scores. The Spotter task was not included in the analysis because this task was the only one that underwent major modifications, and it did not make sense to compare its modified version with its original version at all.

A multiple linear regression model was fitted to predict the scores on each performance metric using sociodemographic variables, device label, and task modifications label as predictors. Sociodemographic variables and device label were included in the regression models as potential confounding factors. The reference category for task modifications was set to be the original version of the task. The effect size of task modifications was quantified as the beta coefficient obtained from the regression model divided by the standard deviation of the predicted performance metric. Two separate regression models were fitted for each cognitive task, one for accuracy and one for response time. The significance of the resulting standardised regression coefficients was assessed using a series of t-tests with an alpha threshold of 0.05.

As presented in Fig. S7, task modifications were not a significant predictor of performance across most metrics. Furthermore, where task modifications did have a significant impact on performance, the effect sizes were generally very small ($\beta < 0.1$) and thus considered negligible. The exceptions were Verbal Analogies and Manipulations 2D, for which task modifications exhibited a medium and small effect on performance accuracy, respectively. Nonetheless, these were the only tasks that underwent modifications beyond just the orientation of presentation.

S3.3: Normative data for modified tasks

All GBIT timepoints, except for the last follow-up, used the original version of the tasks. However, not every task for which normative data were required in Stages 2 and 3 was included in this final GBIT timepoint, leading to a lack of normative data for the modified version of some tasks. To address this, the impact of task modifications on performance was evaluated across tasks. This was tested using patient data, after merging the two available timepoints, because it was the only way to have a dataset containing both versions of each task (before and after modifications). It was assumed that the control data would show a similar effect. For the tasks where only orientation was modified, the impact on performance was either

non-significant or very small; hence it was deemed negligible, allowing the use of control data obtained performing horizontal tasks as normative for patient data obtained performing vertical tasks. This approach was chosen also to: i) maintain consistency with what was done in Stage 1 (same normative data where possible), allowing for a fair comparison between patient timepoints; ii) have larger normative data as more observations were available for the horizontal version of the tasks which were used in most GBIT timepoints. For Verbal Analogies, significant performance differences were observed due to task modifications related to how analogies of varying difficulty were sampled. However, the modified version of this task was included in the final GBIT timepoint, providing relevant normative data. In contrast, Spotter underwent major modifications and was not included in the final GBIT timepoint, leading to its exclusion from Stage 2 and 3 analyses pending future control data availability. For Manipulations 2D, trial-by-trial normative data from the original version of the task were cut to match the modified task duration. Primary performance metrics were recalculated for this shorter version of the original task and used to evaluate norms. Despite these adjustments, the task modifications still had a significant, albeit small, impact on performance, necessitating caution in results interpretation. Finally, for Objects Memory Immediate and Delayed, which were introduced in Stage 2 and directly in their vertical versions, data from the final GBIT timepoint including the vertical version of both tasks were utilised as normative.

Note S4: Online vs in-person assessment of cognitive function

S4.1: Stage 3 study procedures

The study was conducted at Charing Cross Hospital through Imperial College London and led by neuropsychologist Elisa Carta and PhD student Alexandra Moura. We recruited 31 people with MS via email among a pool of individuals who had clinically consented to be part of the UK MS Register but had not signed up for the online portal. Participants attended an in-person visit to Charing Cross Hospital to complete two cognitive assessments: a battery of online computerized tasks on a tablet (Samsung Galaxy Tablet or Apple iPad 9th generation) and a standard pen and paper neuropsychological battery administered by an examiner. Elisa was the examiner for the first 11 participants and Alexandra was the examiner for the remaining 20 participants. The first 11 participants used the Samsung Galaxy Tablet, while the remaining 20 used the Apple iPad. To avoid bias from order effects (e.g., fatigue or false memory), the assessments order was alternated between participants. 16 participants performed the neuropsychological battery first, and 15 the online battery. All participants completed both assessments. In total, 22 tests were performed by all participants during their in-person visit.

The online battery was performed on the Cognitron website and included the same 14 tasks used in Stage 2 (Table S13) excluding Spotter. This task was left out of the assessment because we did not have normative data for it at the time. Consequently, we decided not to overload the participants with a task requiring sustained attention.

The neuropsychological battery included 8 tests (considering the Immediate and Delayed versions of CVLT-II and BVMT-R as one) assessing the cognitive domains of learning, memory, information processing speed, language, executive functions and visuospatial processing. 6 of these tests were selected from the Minimal Assessment of Cognitive Function in MS (MACFIMS), a 7-test widely recommended battery for comprehensive multidomain cognitive assessment in MS[1]. The Paced Auditory Serial Addition Test (PASAT) was excluded for two reasons: i) it required a tape recording of digits which we did not have, and ii) people with MS often find the test stressful and frustrating[2]. The Victoria Stroop and Trail Making tests were added because the online battery featured computerised adaptations of them—Switching Stroop and Trail Making. Moreover, they augmented the executive functions component of the MACFIMS.

Finally, the Nine-Hole Peg Test (9HPT) was administered to gauge hand motor function. This test was included in the assessment for two reasons: i) to determine if the online battery was accessible to individuals with hand motor impairment, and ii) to compare with and validate the Motor Control task, which is part of the online battery and was designed specifically to measure hand motor function. While participants completed two trials of the 9HPT with each hand, we did not know which hand they used for the Motor Control task. We assumed they used their "less impaired hand" and therefore compared response time on the Motor Control task with the average response time across trials of the less impaired hand on the 9HPT.

S4.2: Norming of neuropsychological tests

After data collection, the neuropsychological tests were scored for all 31 participants, and the raw scores were compiled into an Excel table. Raw scores were then converted to standardised z-score, adjusting for sociodemographic factors known to affect test performance. To do so, we sourced normative data from the literature, prioritising regression-based norms, larger and more recent normative samples, and consistency across tests where possible. Table S16 details the normative data and source paper used for each test, along with the sociodemographic factors accounted for in each normative model. For most tests, we used regression-based US norms from Parmenter et al. 2010[3], which cover all MACFIMS tests. However, for the DKEFS, we opted for norms from the manual (Delis et al. 2001[4]) as the Parmenter data were based on two card sets, whereas we used only one.

Supplementary References

1. Benedict, R. H. B. *et al.* Minimal neuropsychological assessment of MS patients: A consensus approach. *Clinical Neuropsychologist* **16**, 381–397 (2002).

2. Langdon, D. W. *et al.* Recommendations for a brief international cognitive assessment for multiple sclerosis (BICAMS). *Multiple Sclerosis Journal* **18**, 891–898 (2012).

3. Parmenter, B. A., Testa, S. M., Schretlen, D. J., Weinstock-Guttman, B. & Benedict, R. H. B. The utility of regression-based norms in interpreting the minimal assessment of cognitive function in multiple sclerosis (MACFIMS). *Journal of the International Neuropsychological Society* **16**, 6–16 (2010).

4. Delis, D. C., Kaplan, E. & Kramer, J. H. *Delis-Kaplan Executive Function System (D-KEFS) Manual*. (Psychological Corporation, San Antonio, TX, 2001).
