# Supplementary material for: Large-scale online assessment uncovers a distinct Multiple Sclerosis subtype with selective cognitive impairment
Source: Nat Commun. 2025 Sep 3;16:6938. doi: 10.1038/s41467-025-62156-4 (PMC12408825; doi:10.1038/s41467-025-62156-4)
Supplement: Supplementary file 2 — Reporting Summary [file 41467_2025_62156_MOESM2_ESM.pdf]

nature portfolio

Corresponding author(s): Annalaura Lerede, Richard Nicholas, Adam Hampshire

Last updated by author(s): Nov 14, 2024

Reporting Summary

Nature Portfolio wishes to improve the reproducibility of the work that we publish. This form provides structure for consistency and transparency in reporting. For further information on Nature Portfolio policies, see our Editorial Policies and the Editorial Policy Checklist.

Statistics

For all statistical analyses, confirm that the following items are present in the figure legend, table legend, main text, or Methods section.

n/a	Confirmed	
☐	☒	The exact sample size (*n*) for each experimental group/condition, given as a discrete number and unit of measurement
☐	☒	A statement on whether measurements were taken from distinct samples or whether the same sample was measured repeatedly
☐	☒	The statistical test(s) used AND whether they are one- or two-sided<br>*Only common tests should be described solely by name; describe more complex techniques in the Methods section.*
☐	☒	A description of all covariates tested
☐	☒	A description of any assumptions or corrections, such as tests of normality and adjustment for multiple comparisons
☐	☒	A full description of the statistical parameters including central tendency (e.g. means) or other basic estimates (e.g. regression coefficient) AND variation (e.g. standard deviation) or associated estimates of uncertainty (e.g. confidence intervals)
☐	☒	For null hypothesis testing, the test statistic (e.g. *F*, *t*, *r*) with confidence intervals, effect sizes, degrees of freedom and *P* value noted<br>*Give P values as exact values whenever suitable.*
☐	☒	For Bayesian analysis, information on the choice of priors and Markov chain Monte Carlo settings
☐	☒	For hierarchical and complex designs, identification of the appropriate level for tests and full reporting of outcomes
☐	☒	Estimates of effect sizes (e.g. Cohen's *d*, Pearson's *r*), indicating how they were calculated

Our web collection on statistics for biologists contains articles on many of the points above.

Software and code

Policy information about availability of computer code

Data collection	Cognitive data were collected using the Cognitron platform embedded within the UK MS Register website which register members were more familiar with. Cognitron tasks included in the study can be tried at this link: https://testingsite.cognitron.co.uk/. The sociodemographic questionnaire was also collected via the Cognitron platform. The questionnaire was administer before the task battery to ensure all participants completed it.<br><br>The patient-reported outcomes included in the study were collected on the UK MS Register website via REDCap.
Data analysis	Data analysis was conducted in Python (3.11.7). The main libraries used were NumPy (1.26.4), pandas (2.1.4), statsmodels (0.14.0), SciPy (1.11.4), scikit-learn (1.2.2), factor-analyzer (0.5.1), and scikit-posthocs (0.9.0). Visualisation were created with matplotlib (3.8.0), seaborn (0.13.2), and PowerPoint.

For manuscripts utilizing custom algorithms or software that are central to the research but not yet described in published literature, software must be made available to editors and reviewers. We strongly encourage code deposition in a community repository (e.g. GitHub). See the Nature Portfolio guidelines for submitting code & software for further information.

Data

Policy information about availability of data

All manuscripts must include a data availability statement. This statement should provide the following information, where applicable:

- Accession codes, unique identifiers, or web links for publicly available datasets
- A description of any restrictions on data availability
- For clinical datasets or third party data, please ensure that the statement adheres to our policy

All data in this study are maintained in a Trusted Research Environment (TRE) which is subject to rules related to access, security, and disclosability of data. All researchers requesting access to data must apply via, and be approved by, the UK MS Register Scientific Steering Committee (SSC), have completed the MRC GDPR training course, and be reputable researchers from a recognised academic institution. The SSC meets quarterly and (if approved) access to the data can be given within 30 days. Our participants' consent is given contingent upon data not being released from the TRE, due to the possibility of linking data from this dataset to extant datasets and the potential for it to become disclosive. For inquiries regarding data access, please contact Dr Rod Middleton at r.m.middleton@swansea.ac.uk.

Research involving human participants, their data, or biological material

Policy information about studies with human participants or human data. See also policy information about sex, gender (identity/presentation), and sexual orientation and race, ethnicity and racism.

Reporting on sex and gender	In the questionnaire at the beginning of the assessment, we asked participants about their sex and we asked them to choose between three options: "Male", "Female" and "Other". Therefore, sex was determined based on a self-reported method. In Stage 1, 2359 (77.4%) responded "Female", 682 (22.4%) "Male" and 7 (0.2%) "Other". In Stage 2, 2099 (78%) responded "Female", 589 (21.9%) "Male" and 2 (0.1%) "Other". In Stage 3, 23 responded "Female" (74.2%) and 8 (25.8%) "Male". In Stage 3 the examiner could confirm the sex with the participant. The higher prevalence of female is reflective of the MS population composition. Sex was accounted for when evaluating adjusted cognitive performance scores. Additionally, we investigated associations between sex and response, completion and return rates to assess whether online cognitive assessment was more feasible/accessible based on sex. Finally, we looked at association between sex and symptom-based cluster and found a sex-related susceptibility to more severe cognitive deficits.
Reporting on race, ethnicity, or other socially relevant groupings	Participants were asked about their age, gender, dominant hand, education, ethnicity, first language, residence and occupation in the questionnaire at the beginning of the assessment (self-reported information). Full questionnaire transcript and resulting categories for each factor are reported in the Supplementary Information. Information on these factors was asked as they could have an impact on cognitive performance. To ensure a fair and unbiased assessment and detect potential impairment in cognitive function caused by MS, participant scores should be compared to scores from controls with similar sociodemographics. For this reason, we evaluated regression-based norms from the general UK population including all the factors mentioned above and the device being used and we used these norms to derive sociodemographically adjusted scores for people with MS. These scores, which we called deviation from expected scores, represent how much each participants deviates from someone with the same sociodemographics but without MS in standard deviation units.
Population characteristics	Covariates taken into account to evaluate adjusted cognitive performance scores using normative data were age, gender, dominant hand, education, ethnicity, first language, residence, occupation and device used for the assessment. Moreover, we investigated associations between adjusted cognitive performance scores and disease subtype and duration.
Recruitment	Participants were recruited because affected by Multiple Sclerosis and part of the UK MS Register. There is potential for selection bias as these participants are already accustomed to engaged with the register online portal and might consequently find online cognitive assessment technology more accessible. Additionally, the UK MS Register includes both a cohort of unverified people with MS who respond to patient-reported outcomes through the internet portal and a cohort of confirmed people with MS for whom clinical data are available and reported by MS Specialist Treatment centres across the UK. Nonetheless, the online cohort has proven to be a representative sample of people with MS in the UK and can be treated as a valid cohort of people with MS also in the absence of supporting clinical validation (Middleton et al. 2018: "Validating the portal population of the United Kingdom Multiple Sclerosis Register").
Ethics oversight	Ethical approval for this study was covered under the UKMSR ethics framework. The UKMSR received ethical approval from the South-West Central Bristol National Research Ethics Service, initially as 16/SW/0194, and subsequently 21/SW/0085. All participants provided informed consent to be involved in the study.

Note that full information on the approval of the study protocol must also be provided in the manuscript.

Field-specific reporting

Please select the one below that is the best fit for your research. If you are not sure, read the appropriate sections before making your selection.

☐ Life sciences ☒ Behavioural & social sciences ☐ Ecological, evolutionary & environmental sciences

For a reference copy of the document with all sections, see nature.com/documents/nr-reporting-summary-flat.pdf

Behavioural & social sciences study design

All studies must disclose on these points even when the disclosure is negative.

Study description	Our study was an observational study carried out over three stages (three data collection timepoints). For Stages 1 and 2 data collection was conducted online. For Stage 3 data collection was conducted in person. We collected both qualitative and quantitative data: sociodemographic and disease-related information via a self-reported online questionnaire (Stages 1,2 and 3), objective cognitive performance data via an online cognitive assessment (Stage 1, 2 and 3) and objective cognitive performance data via a standard neuropsychological assessment administered in person by a trained examiner (Stage 3).<br><br>The online cognitive assessment included a superset of 22 tasks for Stage 1 and a set of 14 tasks for Stages 2 and 3. In Stage 1 participants were administered a 12-task battery including 3 fixed tasks and 9 tasks random sampled from the remaining 19. In Stage 2 and 3 participants were administered an identical 14-task battery in a fixed order. The questionnaire and online cognitive assessment were performed on a personal device remotely in Stages 1 and 2 and on a predisposed tablet in person in the presence of an examiner in Stage 3. Additionally, in Stage 3 participants performed a series of standard neuropsychological tests administered by the examiner. Objective cognitive performance data were derived via automatic scoring for the online cognitive assessment and via manual scoring for the standard neuropsychological assessment.<br><br>Our study also used quantitative patient-reported outcome data collected on a voluntary basis during the UK MS Register periodic (6-monthly) collection windows carried out around the same timeframe of Stage 1 and 2.
Research sample	The research sample is people with MS who consented to be part of the UK MS Register and either signed up for the online portal (Stage 1 and 2) or did not sign up (Stage 3). Demographics of people with Multiple Sclerosis who participated in each stage and of the overall UK MS Register population are reported in the Supplementary Information. The samples for Stage 1 and 2 are representative of the overall MS population as the gender and subtype distributions within these samples are consistent with values reported in the literature and by the Multiple Sclerosis International Federation in their epidemiological report. Additionally, Stage 1 sample does not significantly different from the overall UK MS Register population in terms of age and disease duration.<br><br>The sample for Stage 3 is not representative as it is biased towards younger, more recently diagnosed, and more relapsing remitting people with MS who were more likely to come in the hospital site to receive treatment.
Sampling strategy	For Stage 1 and 2, all valid UK MS Register registrants (with available email, date of birth, gender and subtype at diagnosis) were emailed to take part in the study. Participation was on a voluntary basis. Sample size was not predetermined but the resulting samples were deemed more than sufficient (Stage 1=3,048 and Stage 2=2,690) as bigger than in any other study of this kind carried out in MS. Data collection for Stage 1 and 2 was kept open until no more user responses were recorded for a few days in a row (after reminder emails were sent and response rates were monitored over time).<br><br>For Stage 3, patients from the Hammersmith Hospital site who clinically consented to be part of the UK MS Register but had not signed up on the online portal (N=588) were invited via email to participate in the study. All 31 patients who responded were scheduled for an in person appointment and tested.
Data collection	For Stage 1 and 2, data collection was conducted online. Each participant used a personal device to complete the questionnaire and perform the online cognitive assessment. The assessment could be performed on any personal device with an internet connection, including smartphones, tablets, laptops, or desktop computers, from one's home, without supervision by a trained examiner. Questionnaire responses and cognitive performance scores were recorded via the Cognitron platform, hosted on the UK MS Register website.<br>For Stage 3, data collection was conducted in person. Participants attended an in-person visit in Charing Cross Hospital with an examiner. Elisa Carta was the examiner for the first 11 participants and Alexandra Moura was the examiner for the remaining 20. The questionnaire and the online cognitive battery were completed on a designated tablet in a manner analogous to how they would be performed at home, remotely and without examiner supervision. The first 11 participants used a Samsung Galaxy Tablet, while the remaining 20 used an Apple iPad. The standard neuropsychological battery was administered by the examiner, who recorded responses using pen and paper. Each test was later scored by the examiner based on the recorded responses.
Timing	Stage 1 was carried out between October 2021 and May 2022.<br>Stage 2 was carried out between November 2022 and January 2023.<br>Stage 3 was carried out between January and March 2024.<br><br>Patient-reported outcome data utilised in the study were collected during the UK MS Register periodic collection windows occurring in Autumn 2021, Spring 2022 and Autumn 2022.<br><br>Normative data utilised in the study was collected as part of the Great British Intelligence Test (GBIT) study between December 2019 and February 2023 across 2 baseline timepoints and 4 follow up timepoints.
Data exclusions	Participants <16 or >90 were excluded because we only had normative data in the range between 16 and 90. Additionally, participants who provided invalid responses to, or did not complete, the questionnaire were excluded. This was done because we needed the questionnaire responses to adjust the cognitive performance scores for factors such as age, gender and education that are known to affect cognitive performance and derive deviation from expected scores. Overall, 4 participants were excluded in Stage 1 and 5 in Stage 2.<br><br>Participants task performance records were excluded if non-compliant behaviour was detected. Non-compliant behaviours include people who respond clicking a random answer, resulting in really short response times, or who leave the task open without giving any

response, resulting in the case of timed tasks in null summary accuracy and response time scores. Non-compliant behaviour can be detected by looking at the accuracy and response time distributions for each task, and based on these distributions define thresholds for non-compliance (a min and a max value on the response time distribution and a min value on the accuracy distribution). After excluding non-compliant task performance records, 3,048 participants had valid records on at least 1 task in Stage 1 and 2,690 in Stage 2. Overall, 14 participants were excluded for non-compliant behaviour in Stage 1 and 1 in Stage 2. The number of records excluded for each task in Stage 1 and 2 is reported in the Supplementary Information.

Non-participation

Participation was on a voluntary basis. Of the total 19,188 people with MS emailed, 3,066 participated in Stage 1 and 2,696 in Stage 2. Of the people with MS who participated, 661 did not complete the online cognitive assessment in Stage 1 and 499 in Stage 2. The overall response rates were 16% in Stage 1 and 14% in Stage. These values compared favourably to the rates normally achieved by the UK MS Register, with 17% of the total register users having completed all core patient-reported outcomes during the collection windows around the timeframe of the study. Completion rates were 78.4% in Stage 1 and 81.5% for Stage 2. These values compared favourably to other studies using the Cognitron in an older adult birth cohort and in a UK general population epidemiological cohort. In Stage 3, of the 588 emailed, 31 responded and were scheduled for an in person appointment (5%). Response rate in this case was lower as we deliberately decided to target people who did not sign up for the online portal to encourage their engagement with it, and the requirement to come in person for a 2 hours appointment was more demanding.

Randomization

In Stage 1, each participant received a different 12-task battery composed by 3 fixed tasks and 9 tasks randomly sampled from a superset of 19 tasks. In Stage 3, the administration order of the C-MS and standard neuropsychological batteries was randomised across participants, with approximately half completing the C-MS battery first and the standard neuropsychological battery second, and the other half in the reverse order.

Reporting for specific materials, systems and methods

We require information from authors about some types of materials, experimental systems and methods used in many studies. Here, indicate whether each material, system or method listed is relevant to your study. If you are not sure if a list item applies to your research, read the appropriate section before selecting a response.

Materials & experimental systems

n/a	Involved in the study
☒	☐ Antibodies
☒	☐ Eukaryotic cell lines
☒	☐ Palaeontology and archaeology
☒	☐ Animals and other organisms
☒	☐ Clinical data
☒	☐ Dual use research of concern
☒	☐ Plants

Methods

n/a	Involved in the study
☒	☐ ChIP-seq
☒	☐ Flow cytometry
☒	☐ MRI-based neuroimaging

Plants

Seed stocks

Report on the source of all seed stocks or other plant material used. If applicable, state the seed stock centre and catalogue number. If plant specimens were collected from the field, describe the collection location, date and sampling procedures.

Novel plant genotypes

Describe the methods by which all novel plant genotypes were produced. This includes those generated by transgenic approaches, gene editing, chemical/radiation-based mutagenesis and hybridization. For transgenic lines, describe the transformation method, the number of independent lines analyzed and the generation upon which experiments were performed. For gene-edited lines, describe the editor used, the endogenous sequence targeted for editing, the targeting guide RNA sequence (if applicable) and how the editor was applied.

Authentication

Describe any authentication procedures for each seed stock used or novel genotype generated. Describe any experiments used to assess the effect of a mutation and, where applicable, how potential secondary effects (e.g. second site T-DNA insertions, mosiacism, off-target gene editing) were examined.
